# The expression characteristics of miR-206-3p in musculoskeletal tissue and its clinical significance

**DOI:** 10.1186/s13018-026-06785-5

**Published:** 2026-03-25

**Authors:** Khan Akhtar Ali, Xuefeng Yuan, Tianxiang Cui, Qianqian Yu, Zhiqian Yi, Hui Huang

**Affiliations:** Department of Orthopedics, Tongji Hospital, Tongji Medical College, Huazhong University of Science and Technology, Wuhan, 430030 Hubei China

**Keywords:** miR-206-3p, Osteosarcopenia, Single-cell transcriptomics, Muscle satellite cells, Osteoprogenitor cells, Bone-muscle axis

## Abstract

**Background and objective:**

Osteosarcopenia, a comorbidity of osteoporosis and sarcopenia in the elderly, involves bone-muscle crosstalk, but its core molecular mechanism remains unclear. miR-206 is traditionally considered muscle-specific; this study explores miR-206-3p’s expression in musculoskeletal tissue and correlation with clinical parameters.

**Methods:**

A prospective cohort of 158 elderly hip fracture patients (79 osteosarcopenia, 79 controls) was enrolled. qRT-PCR, in situ hybridization, and scRNA-seq were used to analyze miR-206-3p’s expression, distribution, and cell specificity. Correlations with grip strength, gait speed, and BMD were assessed.

**Results:**

miR-206-3p was significantly downregulated in both tissues of the osteosarcopenia group (P < 0.001), highly expressed in Myod1⁺ muscle satellite cells and Runx2⁺ osteoprogenitor cells (> 82%), and weakly in mature cells (< 12%). It positively correlated with grip strength, gait speed, and BMD (r = 0.562–0.682, P < 0.001).

**Conclusion:**

miR-206-3p is co-expressed in bone-muscle progenitor cells, with synchronous downregulation linked to functional decline, challenging its "muscle-specific" notion and serving as a key molecular hub for bone-muscle crosstalk.

**Supplementary Information:**

The online version contains supplementary material available at 10.1186/s13018-026-06785-5.

## Introduction

With the global aging population accelerating, osteosarcopenia defined as the coexistence of osteoporosis and sarcopenia has emerged as a prevalent clinical syndrome that severely compromises the quality of life and functional independence of the elderly [1–7]. Traditional research has long treated the skeletal and muscular systems as independent entities, neglecting their bidirectional crosstalk in development, metabolism, and age-related degeneration [8–14]. Since the proposal of the “bone-muscle axis” in 2008 [15], accumulating evidence has confirmed that bone and muscle communicate through mechanical transduction, endocrine signaling, and paracrine factors [16, 17], but the core molecular regulators orchestrating this network remain incompletely elucidated [18–20].

Non-coding RNAs (ncRNAs), particularly microRNAs (miRNAs), have emerged as pivotal epigenetic regulators in musculoskeletal homeostasis and disease. Their roles span multiple musculoskeletal conditions: miRNAs modulate tendon healing by regulating tenocyte proliferation and extracellular matrix (ECM) remodeling [21]; in osteoarthritis (OA), dysregulated miRNAs contribute to chondrocyte dysfunction and cartilage degradation, with meta-analyses confirming their high diagnostic accuracy (AUC = 0.90) [22, 23]; small interfering RNAs (siRNAs) target key genes to influence tendon homeostasis and osteoporosis progression [24, 25]; and circular RNAs (circRNAs) have been implicated in osteoporosis management [26]. Notably, ncRNAs act as inter-tissue messengers, mediating crosstalk by targeting shared signaling pathways (e.g., Wnt/β-catenin, OPG/RANKL/RANK) [27, 28], highlighting their potential as integrated regulators of the bone-muscle axis across diverse musculoskeletal disorders.

miR-206 has long been classified as a “muscle-specific miRNA,” primarily studied for its role in muscle satellite cell proliferation and differentiation [9, 10]. However, recent studies challenge this narrow classification: in vitro experiments demonstrate that miR-206 inhibits osteogenic differentiation by targeting Glutaminase (GLS) [6], while clinical data link miR-206-3p expression in bone tissue to bone mineral density (BMD) [8]. Parallel to this, other miRNAs exhibit similar dual-tissue regulation: miR-217 modulates bone metabolism via the OPG/RANKL/RANK pathway in postmenopausal osteoporosis (PMOP) [28]; miR-106a-5p regulates osteoblast function through the PTEN axis in PMOP [29]; and miR-204-5p protects against intervertebral disc degeneration (IVDD) by targeting the SSRP1/NF-κB pathway [30]. These findings suggest that miR-206-3p may belong to a broader class of miRNAs with pleiotropic roles in musculoskeletal homeostasis.

Clinically, osteosarcopenia diagnosis relies on validated tools such as the SARC-F questionnaire and handgrip strength measurements [13]. Recent studies underscore the utility of these tools in diverse populations [31, 32], but their molecular correlates remain poorly defined. Meanwhile, miRNAs have emerged as promising non-invasive biomarkers for musculoskeletal diseasesYang et al. [23] reported that miRNAs exhibit 81% sensitivity and 85% specificity for OA diagnosis, and serum lncRNA CRNDE effectively predicts delayed fracture healing [33, 34]. These findings support the need to identify molecular biomarkers that complement clinical tools for osteosarcopenia.

This study enrolled three groups: 79 osteosarcopenia patients (hip fracture + osteoporosis + sarcopenia), 79 fracture-matched controls (hip fracture without osteosarcopenia), and 79 age-matched healthy controls. We integrated qRT-PCR, in situ hybridization, single-cell RNA sequencing (scRNA-seq), and preliminary luciferase assays to systematically investigate miR-206-3p’s expression profile, cell-type specificity, and correlation with clinical phenotypes (SARC-F score, grip strength, BMD). Our objectives were to: (1) challenge the “muscle-specific” paradigm of miR-206; (2) explore its role as a regulatory node in bone-muscle crosstalk, drawing parallels to ncRNA-mediated regulation in OA, osteoporosis, and tendon injuries [21, 22, 25]; and (3) evaluate its utility as a complementary biomarker to clinical screening tools, building on the diagnostic potential of miRNAs in musculoskeletal diseases [23, 29]. Functional validation of its regulatory mechanisms will be addressed in subsequent in vitro experiments.

## Materials and methods

### General information

This study prospectively collected clinical samples of elderly fracture patients who met the inclusion criteria from the Orthopedics Department and Geriatrics Department of our hospital between November 2022 and December 2023. All patients signed Informed consent after admission and before surgery. The study protocol was reviewed and approved by the Ethics Committee of the hospital (Approval No: TJ-IRB20221128/TJ-IRB202512049) and conducted in strict accordance with the ethical principles of the Declaration of Helsinki.

The inclusion criteria were as follows: (1) Aged 65 years or older; (2) Suffered a hip or proximal femur fracture due to low-energy trauma (such as a fall), with surgical indications (both the sarcopenia group and the control group consisted of such patients); (3) An additional 79 age-matched healthy elderly volunteers (no fracture history, bone density T-score > -1.0, and normal muscle function) were included as healthy controls to verify the differential expression of miR-206-3p in disease and healthy states; (4) The patient/volunteer or their legal representative agreed to provide tissue samples (tissue obtained during/after surgery for fracture patients, and a small amount of normal tissue obtained from minimally invasive procedures in the Orthopedics Department for healthy controls); (5) Able to cooperate with the completion of grip strength and gait speed assessments, the SARC-F questionnaire, and DXA bone densitometry.

Exclusion criteria included: (1) suffering from other metabolic bone diseases besides osteoporosis (such as hyperparathyroidism, osteogenesis imperfecta, etc.); (2) suffering from known primary or secondary myopathies (such as polymyositis, myasthenia gravis, etc.); (3) having a history of neoplasm malignant or receiving radio chemotherapy within the past 6 months; having severe heart, liver, or kidney dysfunction (defined as NYHA cardiac function class III-IV, Child–Pugh class C, or eGFR 3 months) use of drugs affecting bone metabolism or muscle metabolism (such as Glucocorticoids, aromatase inhibitor, statins, etc.); (6) those with seriously Deletion of clinical data or follow-up data.

The diagnostic criteria for the sarcopenia group were patients who simultaneously met the criteria for osteoporosis (determined by DXA, T-score ≤ -2.5) and sarcopenia (according to the Asian Working Group for Sarcopenia AWGS 2019 criteria, i.e., grip strength -1.0 and muscle function indicators (grip strength, gait speed) within the normal range.

Finally, a total of 79 patients were included in the sarcopenia group, 79 patients were included in the control group (non-osteosarcopenia fracture), and 79 patients were included in the healthy control group; the sample size was determined by G*Power 3.1 based on the effect size of previous osteosarcopenia miRNA studies (miR-206-3p expression difference fold change = 1.8, α = 0.05, β = 0.2) to ensure a statistical power ≥ 80%.

The sarcopenia group had an average age of (76.41 ± 5.23) years, with 35 males (44.30%). Patients in this group generally exhibited characteristics such as low Weight, decreased BMI, significantly reduced grip strength and gait speed, elevated Strength, Assistance with walking, rising from a chair, climbing stairs, falls scores, and severely reduced bone mineral density, accompanied by a higher incidence of comorbidities such as diabetes and hypertension. The control group had an average age of (74.58 ± 4.87) years, with 39 males (49.37%). Patients in this group had normal or slightly higher levels of Weight, Body height, and BMI, good functional indicators such as grip strength and gait speed, low Strength, Assistance with walking, rising from a chair, climbing stairs, Falls scores, bone mineral density indicators within the normal range, and a relatively low incidence of comorbidities.

To further clarify the miR-206-3p's prognostic value in different clinical subgroups, this study stratified 158 elderly hip fracture patients by fracture type (Tile A/B/C) and age (65–74 years vs. ≥ 75 years).Among them, 42 cases (26.6%) were Tile A fractures, 68 cases (43.0%) were Tile B, and 48 cases (30.4%) were Tile C; 83 patients (52.5%) were 65–74 years old, and 75 patients (47.5%) were ≥ 75 years old. All patients completed a 1-year follow-up, with 6-month fracture delayed union (defined as insufficient callus formation or persistent interfragmentary gap on imaging) and re-fall events within 1 year (confirmed by outpatient or telephone follow-up) being recorded.

### Experimental method

Tissue sample collection and processing: All samples were obtained when patients underwent internal fixation of fracture surgery. After incising and exposing the fracture end, approximately 50–100 mg of muscle tissue biopsy samples were taken from the quadriceps femoris muscle (rectus femoris muscle or vastus lateralis muscle). Simultaneously, approximately 200–300 mg of cancellous bone fragments were collected during burr hole or intramedullary reaming in the proximal femur metaphysis. After collection, the samples were immediately rinsed three times with pre-cooled sterile saline to remove blood stains. Subsequently, a portion of the samples was rapidly frozen in liquid nitrogen and then transferred to a -80 °C freezer for subsequent RNA extraction and qRT-PCR analysis; another portion of the samples was immediately fixed in 4% paraformaldehyde solution for 24–48 h for paraffin wax embedding and in situ hybridization experiments.

qRT-PCR assay: Total RNA was extracted from frozen muscle and Bone tissue using TRIzol reagent… Real-time fluorescence quantitative PCR was performed using the SYBR Green method, with U6 snRNA as the internal reference gene (verified by GeNorm software analysis, the expression stability M value of U6 in Bone tissue = 0.38 < 0.5, significantly better than other candidate internal references such as 5S rRNA (M = 0.62), suitable as an internal reference for Bone tissue miRNA detection), to detect the relative expression level of miR-206-3p. Primer sequences (designed and synthesized by Shanghai Gemma): miR-206-3p forward primer5'-GGAATGTAAGGAAGTGTGTGAT-3',reverse primer 5'-CAGTGCGTGTCGTGGAGT-3'; U6 forward primer 5'-CTCGCTTCGGCAGCACA-3', reverse primer 5'-AACGCTTCACGAATTTGCGT-3'; all primers were verified by BLAST to have no non-specific binding, and the melting curve showed a single peak (Tm value stable), and the amplification efficiency was 95%-105%.

Each sample was set up with 3 replicates, and the experiment was repeated 3 times.

in situ hybridization: The fixed tissue samples were subjected to paraffin wax embedding and sectioned (4 μm thick). Hybridization was performed using a specific Digoxin-labeled Locked Nucleic Acid (LNA) probe for miR-206-3p (sequence: 5'-Dig-AGAGAAGGAAGTGTGTGATCCAA-3', synthesized by Exiqon company, Tm value = 72℃); meanwhile, a scramble LNA probe (sequence: 5'-Dig-ACGTGATCGTACGATCGTACG-3', with no homology to the human genome) was set as a negative control to ensure the specificity of the hybridization signal. After hybridization, signal detection was performed using anti-Digoxin antibody (1:500 dilution, Abcam company) and ALP (ALP) chromogenic system (NBT/BCIP substrate, Sigma company), DAPI was used to counterstain the cell nuclei, and finally observed and photographed under an optical microscope (OlympusBX53) to determine the spatial distribution of miR-206-3p in specific tissue structures such as muscle fibers and bone trabeculae.

Single-cell RNA Sequencing (scRNA-seq): Freshly obtained muscle and bone tissue samples were digested with collagenase and dispase under sterile conditions to prepare single-cell suspensions. Cell viability was assessed by trypan blue staining and counting (requiring > 85% viable cells). Single-cell capture, cDNA library construction, and sequencing were performed using the 10 × Genomics Chromium platform according to its standard protocol (v3.1 reagent kit). Sequencing was performed on the Illumina NovaSeq 6000 platform, targeting > 50,000 reads per cell. In subsequent analyses, "miR-206-3p high expression" was defined as the expression level of this gene in a cell being greater than 1.5 times the median gene expression in all detected cells (i.e., foldchange > 1.5), as determined by the "Find Markers" function of the Seurat package.

### Data processing

The raw sequencing data was generated by the Illumina sequencer. First, Cell Ranger software (version 7.0.0) was used for primary Data processing, including converting BCL files to FASTQ format, performing sequence alignment (reference genome is GRCh38), generating gene expression matrix (feature-barcode matrix), and performing preliminary quality control (such as filtering out cells with excessively high mitochondrial gene ratios or excessively low total UMI counts). Subsequently, the processed data was imported into the R language environment (version 4.2.0), and the Seurat package (version 4.3.0) was used for advanced analysis. Based on Graph-based clustering analysis (resolution parameter set to 0.5): referring to the common resolution range (0.4–0.6) [35] for single-cell studies of musculoskeletal tissue, and verified by pre-experiments (excessive merging of cell clusters at resolution 0.3, redundant small clusters at resolution 0.7), 0.5 was determined as the optimal resolution, which can clearly distinguish target cell types such as muscle satellite cells and osteogenic progenitor cells without over-clustering.

Cell annotation was mainly based on known cell type marker genes, such as Myod1, Myog for muscle satellite cells and myoblasts, Runx2, Sp7 for osteogenic progenitor cells, Col1a1, Spp1 for mature osteocytes, Myh1, Myh2 for mature muscle fiber cells, etc. To track cell differentiation trajectories, pseudo time analysis was performed on Myod1 + and Runx2 + cell populations using the Monocle3 package to reveal the dynamic differentiation process from stem cells to mature cells. In addition, gene Set Enrichment Analysis (GSEA) was performed on cell subpopulations with high expression of miR-206-3p and subpopulations with low expression, using the "Hallmark" gene set in the MSigDB database to assess the activity differences of key signaling pathway such as Wnt/β-catenin.

### Statistical analysis

All statistical analyses were performed using SPSS 26.0 software and R language (version 4.2.0). Measurement data were first verified for normality by the Shapiro–Wilk test (W value > 0.9, P > 0.05 for all indicators, conforming to normal distribution), and expressed as (± s); independent samples t-tests were used for inter-group comparisons. If the data did not conform to the normal distribution, they were expressed as median (interquartile range) [M (Q1, Q3)], and non-parametric tests were used; multiple comparisons among multiple groups were performed using independent samples t-tests combined with Bonferroni correction (such as inter-group comparisons of multiple indicators) to avoid the risk of Type I error. Count data were expressed as number of cases and percentage [n (%)], and inter-group comparisons were performed using the Chi-square test (χ^2^ test) or Fisher's exact test (when the theoretical frequency < 5).

Pearson correlation coefficient (r) was used to assess the linear relationship between miR-206-3p expression levels and clinical indicators. Receiver Operating Characteristic Curve (ROC) was used to determine the optimal cut-off values of miR-206-3p expression levels for predicting “6-month fracture delayed union” and “1-year risk of recurrent fall” (maximum Youden index). Internal validation of ROC-derived cut-off values was performed using 1000 bootstrap samples to assess the stability of the cut-offs and reproducibility of AUC values (bootstrap-corrected AUC and 95% CI were reported to confirm robustness).

Based on the ROC-derived cut-off value, patients were divided into the “miR-206-3p high expression group” and the “low expression group”. For multivariate logistic regression analysis:

1Model-building strategy: Variables were initially selected based on ① univariate analysis results (variables with P < 0.2: age, fracture type, BMI, diabetes, hypertension, osteosarcopenia status) and ② osteosarcopenia-related factors with clear clinical significance (refer to AWGS 2019 diagnostic criteria [13]. A backward elimination method was used for model refinement, with variables retained in the final model if they met P < 0.05.

Handling of potential overfitting: The Hosmer–Lemeshow goodness-of-fit test was used to evaluate model calibration (P > 0.05 indicating no significant lack of fit). Additionally, the C-statistic (AUC) of the final model was compared with that of a reduced model (excluding miR-206-3p) to ensure the model did not overfit to noise.

The study incorporated age, fracture type (with Tile A as the reference), BMI, diabetes, hypertension, and sarcopenia status as covariates to assess the independent association between miR-206-3p expression and adverse prognostic events. All tests were conducted bilaterally, with P < 0.05 indicating statistical significance.

## Result

### Basic clinical characteristics of the study subjects

**Fig. 1:**
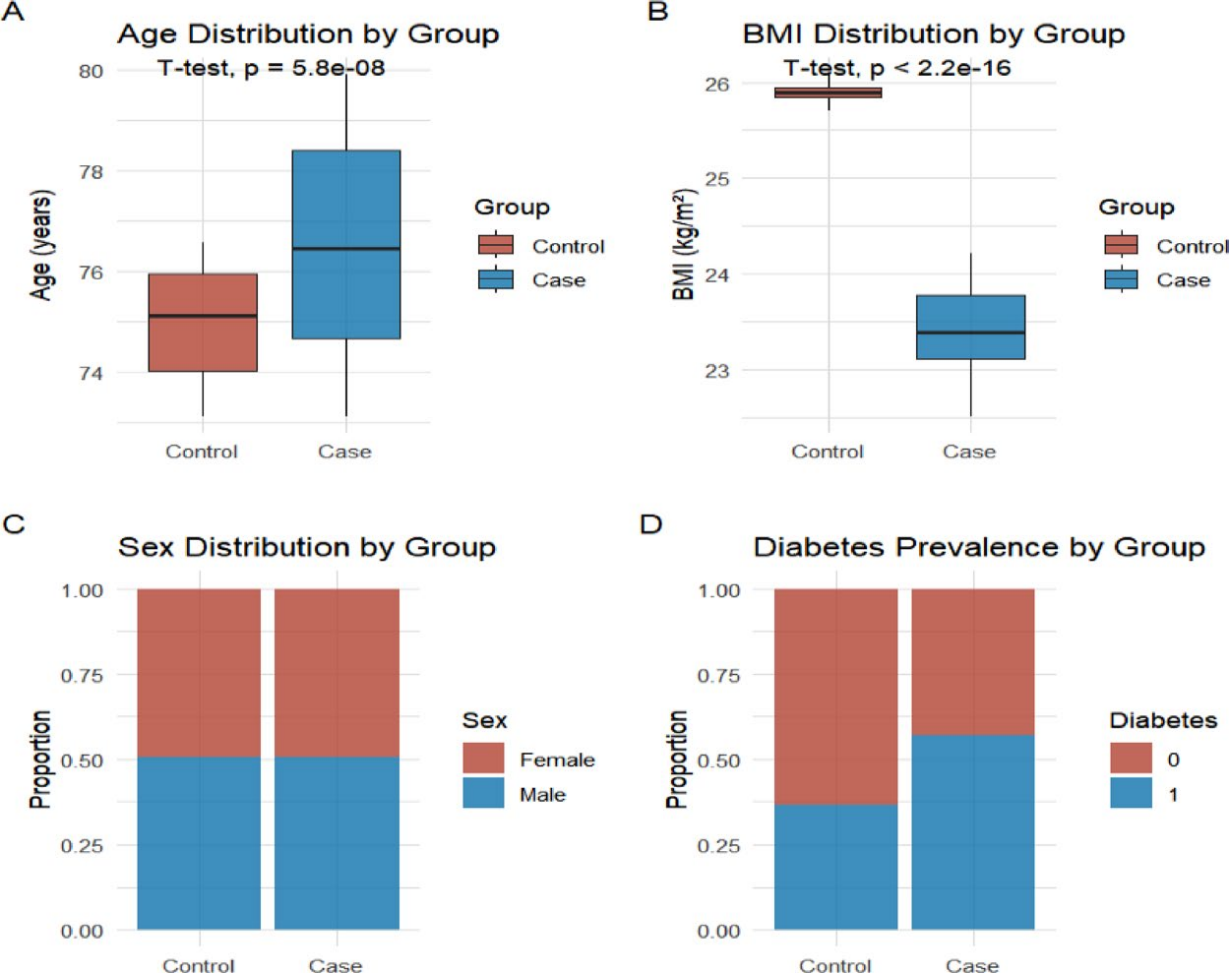
Comparison of case and control groups in demographic and baseline clinical characteristics. **A** Age distribution; **B** Body mass index (BMI) distribution; **C** Gender composition; **D** Diabetes prevalence

**Fig. 2:**
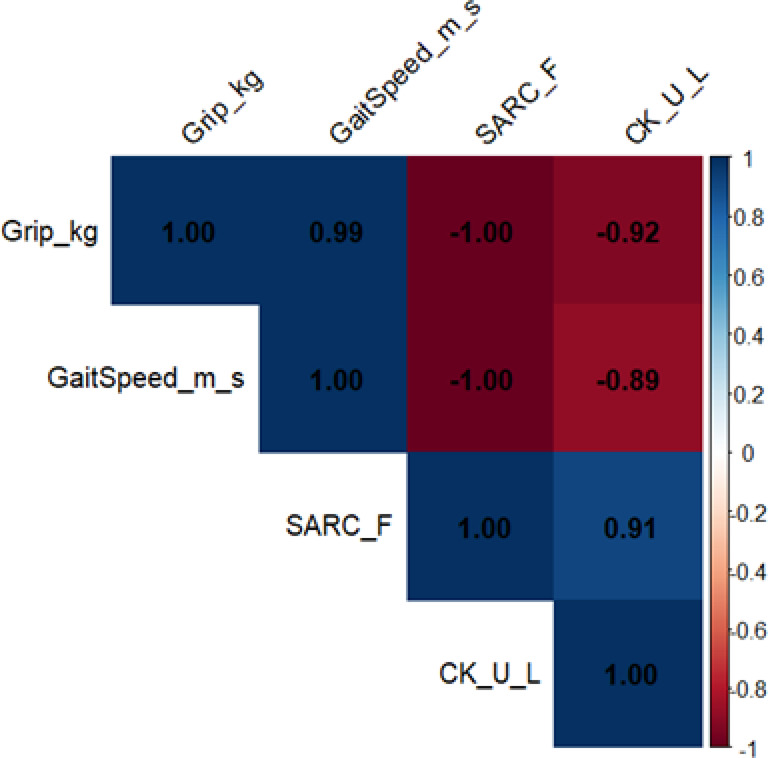
muscle functional indicator correlation matrix. *Note: The four indicators are: grip strength, gait speed, Strength, Assistance with walking, rising from a chair, climbing stairs, Falls questionnaire score, and upper extremity creatine kinase levels

This study included a total of 158 elderly patients over 65 years old, with 79 cases in each of the osteosarcopenia group and the control group (Table 1). This study first compared the demographic and basic traits of the case group and control group (Fig. 1). The average age of the case group was significantly higher, while the BMI level was significantly lower; at the same time, the prevalence of diabetes was significantly increased (all statistically significant), suggesting that it presented typical "Elderly-low nutrition-heavy metabolic burden" trait, which was consistent with the epidemiological pattern of osteosarcopenia-related Illness, laying a reliable population basis for subsequent analysis. Further correlation analysis of muscle function (Fig. 2) showed that there was a significant positive correlation between grip strength and gait speed, and a consistent negative correlation trend with SARC-F scores, while SARC-F was positively correlated with creatine kinase levels, suggesting that the decline in muscle strength is not only consistent with the decline in Motor Abilities, but may also be accompanied by an increased risk of Muscle injury.

**Table 1:** Comparison of basic clinical characteristics between sarcopenia group and control group

Indicator category	Specific indicators	Osteosarcopenia group (n = 79)	Control group (n = 79)	t/χ^2^ value	*P* Value
Demographic characteristics	Age (years)	76.41 ± 5.23	74.58 ± 4.87	2.245	0.026
	Gender (Male) [n (%)]	35(44.30%)	39(49.37%)	0.569	0.451
	Weight (kg)	58.32 ± 8.25	68.74 ± 9.36	6.872	< 0.001^*^
	Hight (cm)	158.24 ± 6.17	162.35 ± 5.82	4.285	< 0.001^*^
	BMI (kg/m^2^)	23.15 ± 2.87	26.03 ± 2.95	5.824	< 0.001^*^
Functional indicators	Grip strength(kg)	18.28 ± 3.17	28.82 ± 4.26	16.651	< 0.001^*^
	Pace (m/s)	0.73 ± 0.14	1.17 ± 0.21	15.174	< 0.001^*^
	SARC-F Score	8.59 ± 1.32	3.27 ± 1.15	28.683	< 0.001^*^
Bone density index	Bone density (g/cm^2^)	0.725 ± 0.123	0.982 ± 0.140	12.444	< 0.001^*^
	Bone mineral density T-score	-2.87 ± 0.52	-0.43 ± 0.38	31.254	< 0.001^*^
	Bone mineral density Z-score	-2.14 ± 0.47	0.28 ± 0.42	32.678	< 0.001^*^
Comorbidity status	Diabetes [n (%)]	28(35.44%)	15(18.99%)	6.237	0.012
	Hypertension [n (%)]	45(56.96%)	32(40.51%)	5.284	0.021
	Cardiovascular disease [n (%)]	22(27.85%)	12(15.19%)	4.376	0.036
Serological indicators	Serum albumin (g/L)	37.45 ± 3.26	41.28 ± 3.54	6.524	< 0.001^*^
	Vitamin D (ng/mL)	18.25 ± 5.36	28.47 ± 6.28	10.245	< 0.001^*^
	Serum IL-6 (pg/mL)	15.32 ± 4.27	8.45 ± 2.36	12.354	< 0.001^*^
	Serum TNF-α(pg/mL)	12.45 ± 3.56	7.28 ± 2.14	10.254	< 0.001^*^
	Serum CRP (mg/L)	8.25 ± 2.36	3.47 ± 1.28	14.254	< 0.001^*^
	Serum IL-16 (pg/mL)	128.45 ± 25.36	85.27 ± 18.54	11.245	< 0.001^*^
Bone Metabolism Markers	Serum osteocalcin (ng/mL)	12.35 ± 3.26	18.47 ± 4.28	9.254	< 0.001^*^
	Serum CTX-I (ng/mL)	0.45 ± 0.12	0.32 ± 0.09	7.854	< 0.001^*^
Muscle Metabolism Biomarkers	serum creatine kinase (U/L)	125.36 ± 35.24	98.47 ± 28.36	4.854	< 0.001^*^

^*^ Inter-group comparisons of quantitative data were performed using t-tests combined with Bonferroni correction, and count data were analyzed using χ^2^ tests combined with Bonferroni correction

^*^P < 0.05 indicates a statistically significant difference after correction

**Fig. 3:**
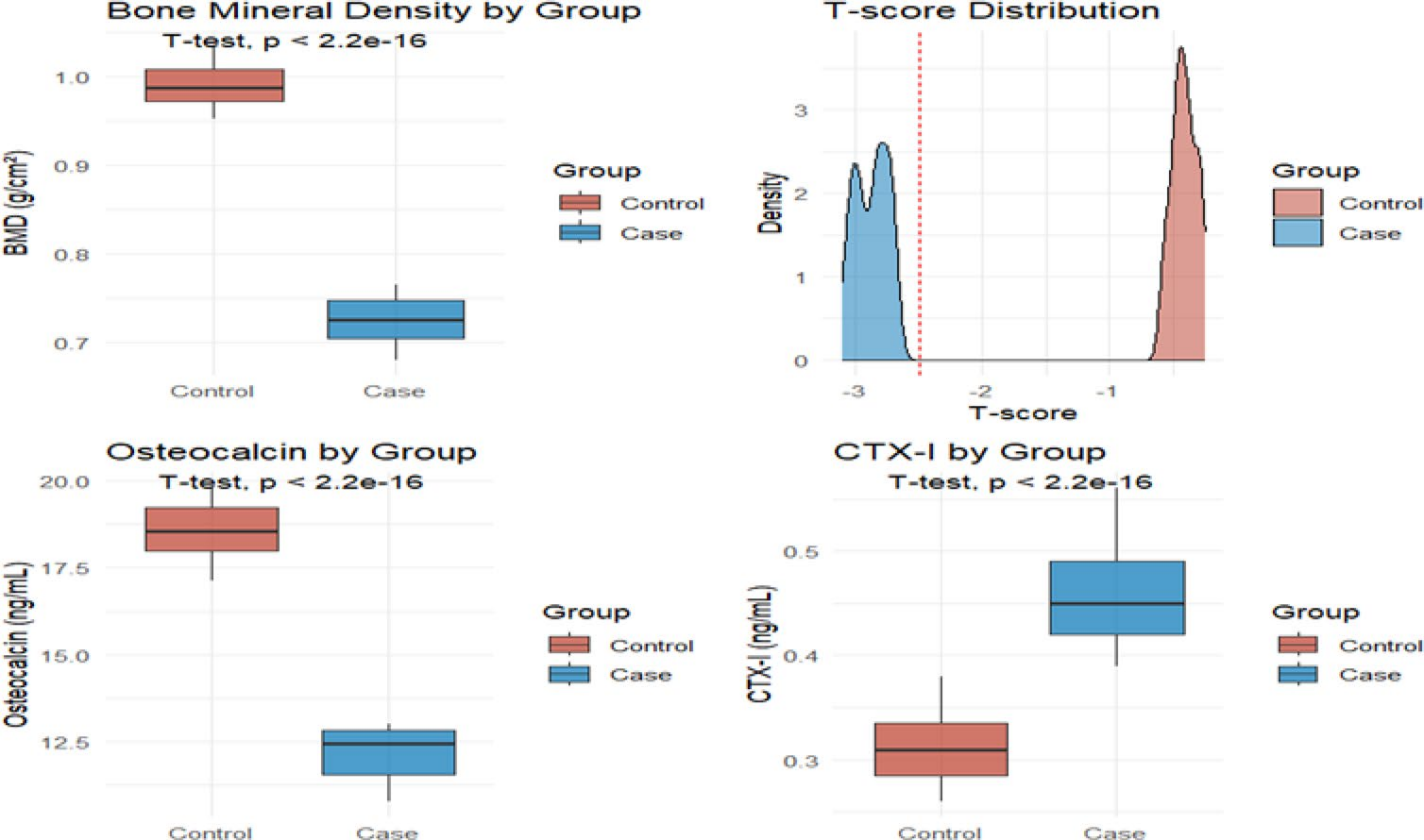
Comparative analysis of three key bone health indicators bone mineral density, osteocalcin, and CTX-I between the case group and the control group

**Fig. 4:**
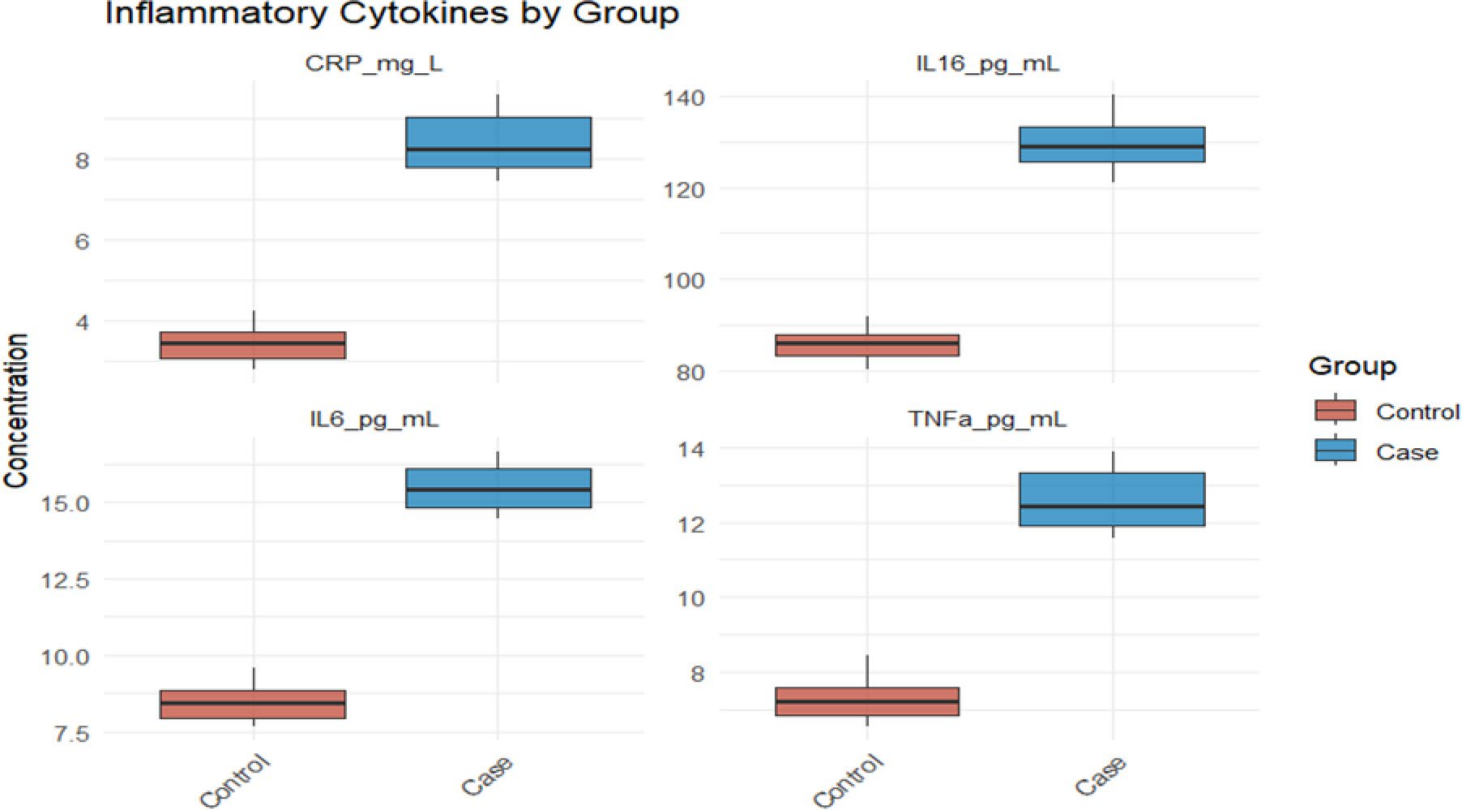
Difference analysis of systemic inflammatory reaction status between the case group and control group

In terms of bone metabolism (Fig. 3), the bone mineral density of the case group was significantly decreased, while the levels of osteocalcin and CTX-I were significantly increased, reflecting that its bone turnover was in an accelerated and bone absorption-dominated abnormal state, which was consistent with the osteoporosis phenotype. The inter-group comparison of inflammation indicators (Fig. 4) showed that CRP, IL-6, IL-8, and IL-10 in the case group were significantly increased, suggesting that it was in a state of sustained systematicity inflammation activation.

**Fig. 5:**
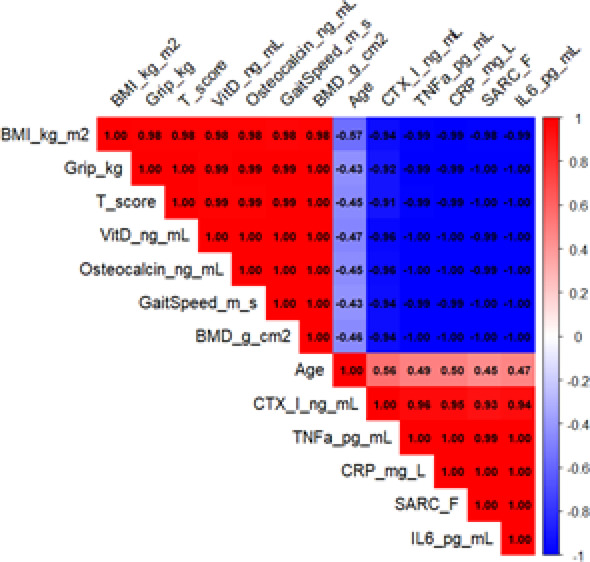
Correlation matrix of clinical parameters. Analyzes correlations among body composition, functional indicators, inflammatory factors, and bone metabolism markers

**Fig. 6:**
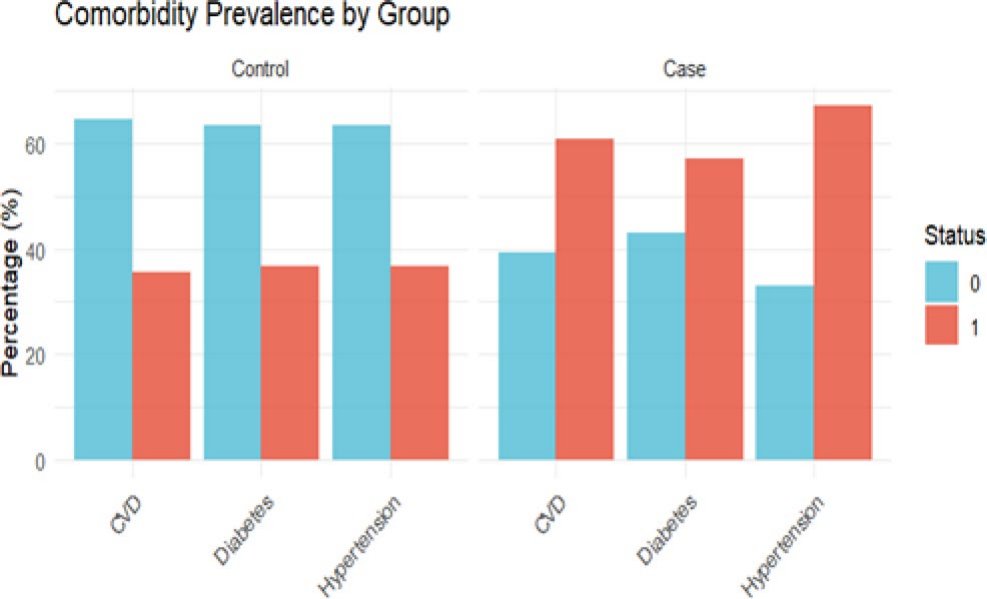
Analysis of prevalence differences between case and control groups in comorbid conditions

Correlation network analysis (Fig. 5) further revealed a clear clustering structure among clinical indicators: BMI, grip strength, Bone mineral density T-score, and gait speed formed a positively correlated "Body composition-bone-function" cluster, while CTX-I, TNF-α, CRP, and Interleukin-6 formed an "Inflammation-bone resorption" cluster; age was negatively correlated with physical fitness indicators and positively correlated with bone resorption indicators, and SARC-F was significantly negatively correlated with functional indicators. These results collectively suggest that inflammation, abnormal bone metabolism, and muscle function decline have synergistic evolutionary traits in the context of aging. Comorbidity analysis (Fig. 6) further revealed that the prevalence of osteoporosis, diabetes, and hypertension was significantly increased in the case group, suggesting that impaired bone health may be closely related to systemic metabolic abnormalities and angiopathy.

**Fig. 7:**
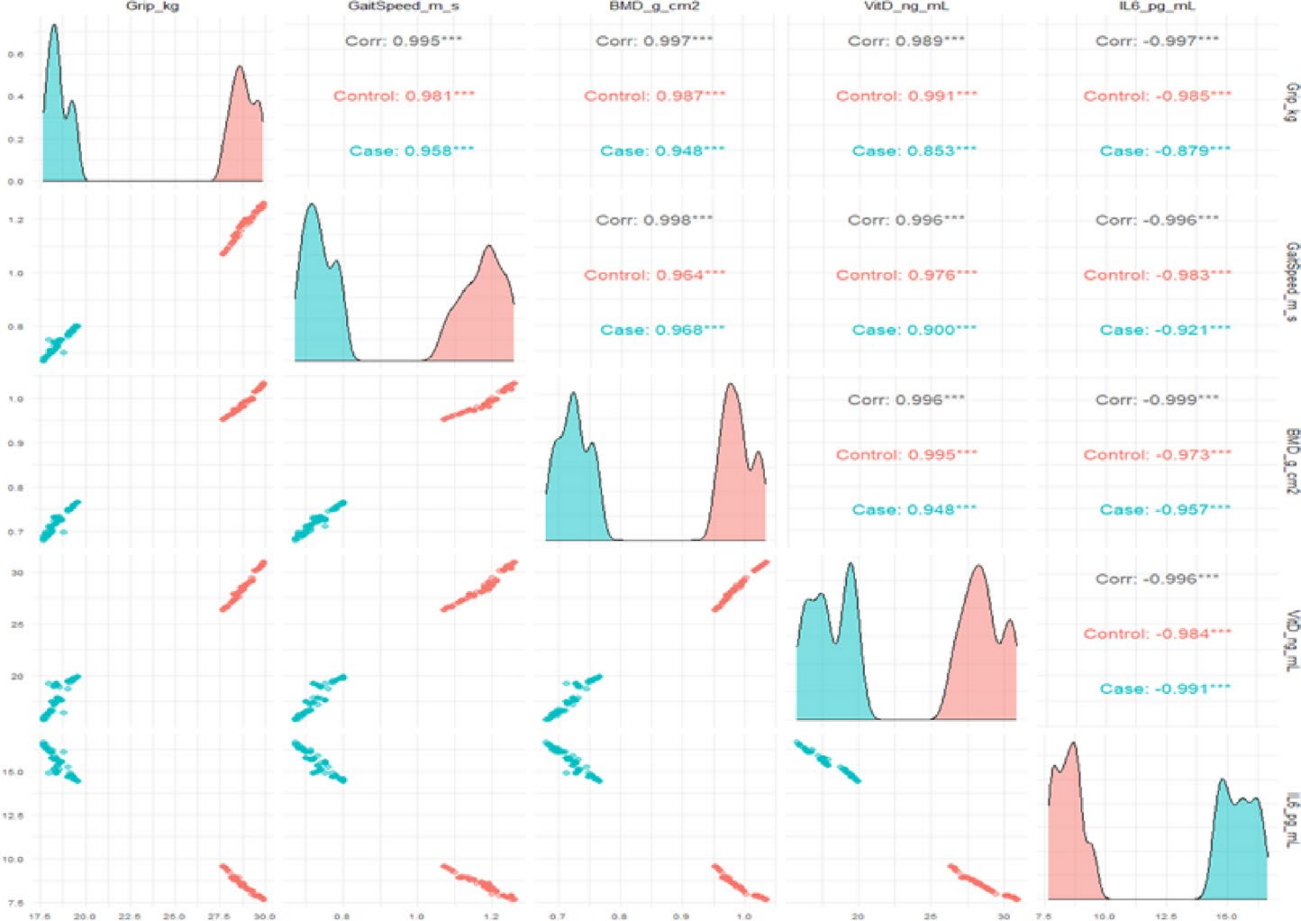
Stratified correlation analysis of the association pattern of muscle skeletal function, Vitamin D levels, and inflammatory factor IL-6

**Fig. 8:**
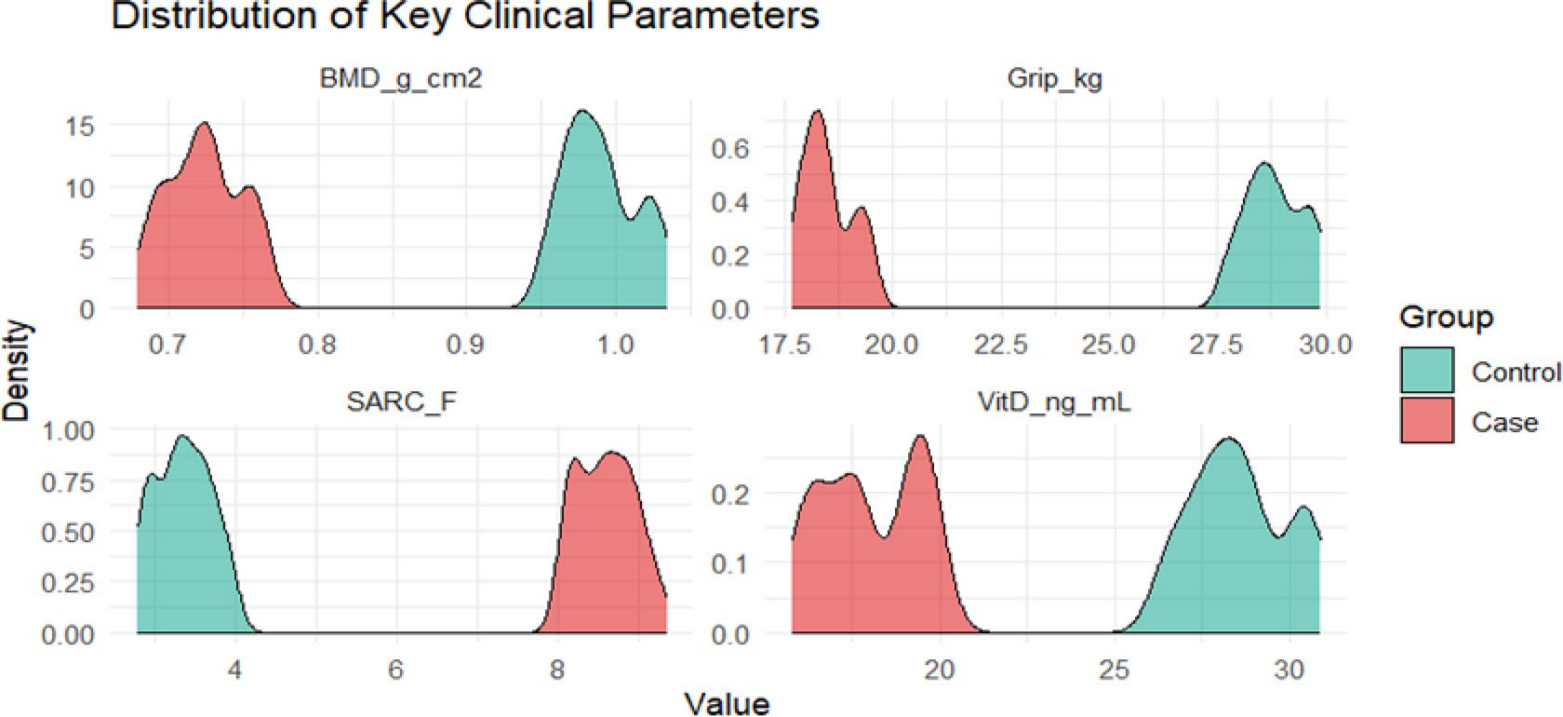
Density distribution of clinical parameters between the case group and control group. Key clinical parameters: bone density, grip strength, walking speed, and vitamin D

Stratified correlation results (Fig. 7) showed that the correlation between Bone-muscle-nutrition indicators in the control group was closer, while the correlation in the case group was weakened as a whole, especially the association between bone mineral density and Vitamin D decreased, suggesting that the body's regulation and linkage ability and homeostasis maintenance function may have been impaired in the disease state. Density distribution analysis (Fig. 8) further verified the above trend from the overall level: the distributions of bone mineral density, grip strength, gait speed, and Vitamin D in the case group were significantly shifted to the left, while the SARC-F distribution shifted to the right, indicating that it showed global decline characteristics in bone health, muscle function, and nutritional status.

### Comparison of miR-206-3p expression levels between the osteosarcopenia group and the control group

**Fig. 9:**
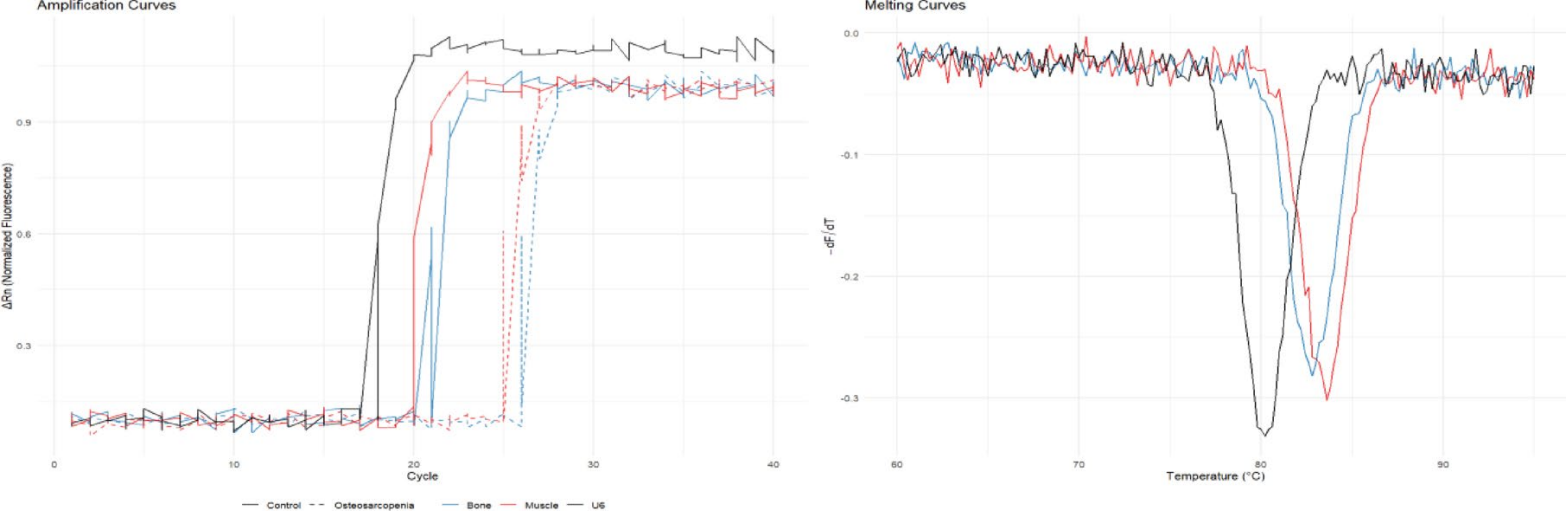
qRT-PCR amplification curves and melting curves of miR-206-3p in skeletal muscle tissue

qRT-PCR results showed that the relative expression level of miR-206-3p in muscle tissue in the osteosarcopenia group was significantly lower than that in the control group; in Bone tissue, the expression level of the osteosarcopenia group was also significantly lower than that of the control group. in situ hybridization further confirmed that miR-206-3p was mainly located around muscle fibers and in the bone trabecular region. The inter-group differences in miR-206-3p expression levels in muscle tissue and Bone tissue were statistically significant (*P* < 0.05), indicating that this miRNA exhibited a synchronous down-regulation trait in both tissues in patients with osteosarcopenia, as shown in Table 2 and Fig. 9.

**Table 2:** Comparison of relative expression levels of miR-206-3p between the Osteomuscular atrophy group and control group

Organization type	Osteosarcopenia group(n = 79)	control group(n = 79)	T Value	*P* Value
Muscle tissue (relative expression level)	1.26 ± 0.31	2.86 ± 0.53	25.752	< 0.001
Bone tissue (relative expression level)	1.09 ± 0.26	2.55 ± 0.47	25.043	< 0.001

**Fig. 10:**
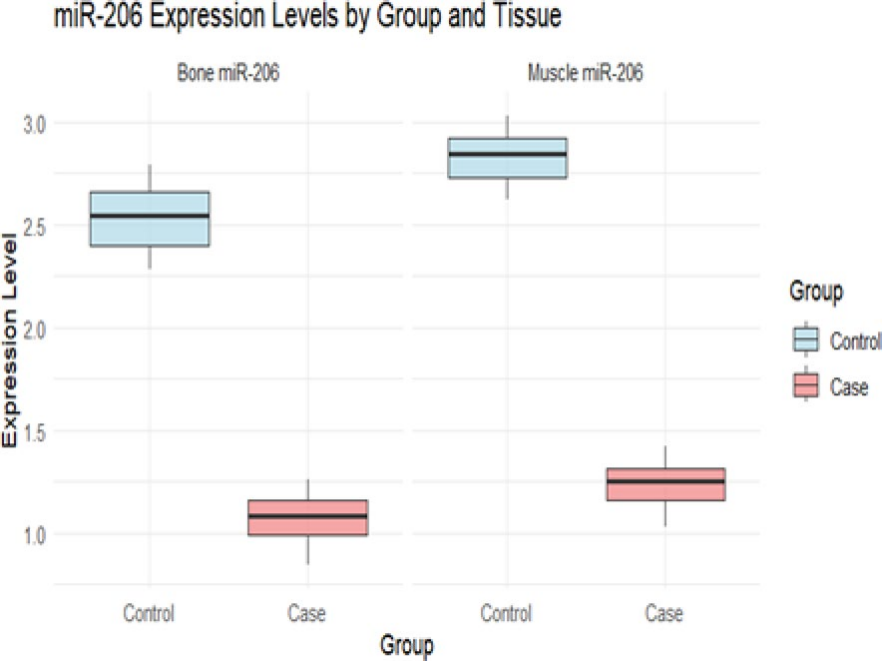
Box plot of miR-206-3p expression differences

**Fig. 11:**
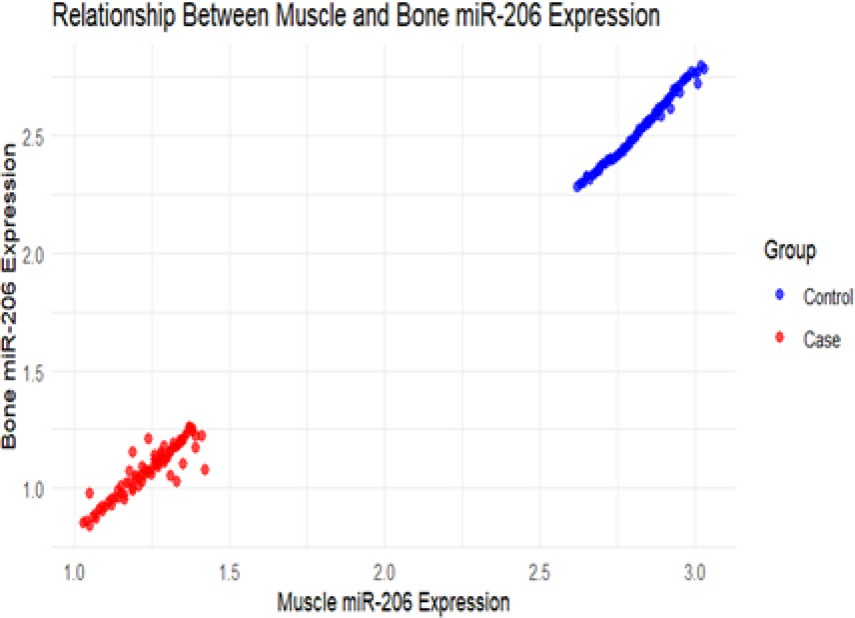
Correlation analysis of miR-206-3p expression levels in muscle and bone tissues

qRT-PCR results showed (Figs. 9) that the relative expression level of miR-206-3p in muscle tissue and bone tissue in the osteosarcopenia group were significantly lower than those in the control group, and the differences had statistical significance (P < 0.05). in situ hybridization results further suggested that this molecule was mainly located around muscle fiber and in the bone trabeculae region, which was consistent with its potential role in exercise and bone metabolism regulation (Table 2, Fig. 1). box plot showed (Fig. 10 Fig. 11) that both groups showed a trend of "muscle tissue expression being higher than bone tissue", but under the disease state, the overall expression of miR-206-3p decreased, and the downregulation amplitude of bone tissue was more significant, suggesting that it was more sensitive to pathological changes.

In addition, scatter plot analysis revealed that the expression levels of miR-206-3p in muscle and bone tissues showed a significant positive correlation, with case group data points mainly clustered in the lower-left quadrant, forming clear inter-group separation characteristics. The above results indicate that miR-206-3p exhibits a pattern of synchronous downregulation in both tissues in osteosarcopenia and may serve as a key molecular marker connecting muscle and bone metabolism Abnormal.

### Expression distribution of miR-206-3p in different cell subpopulations

**Fig. 12:**
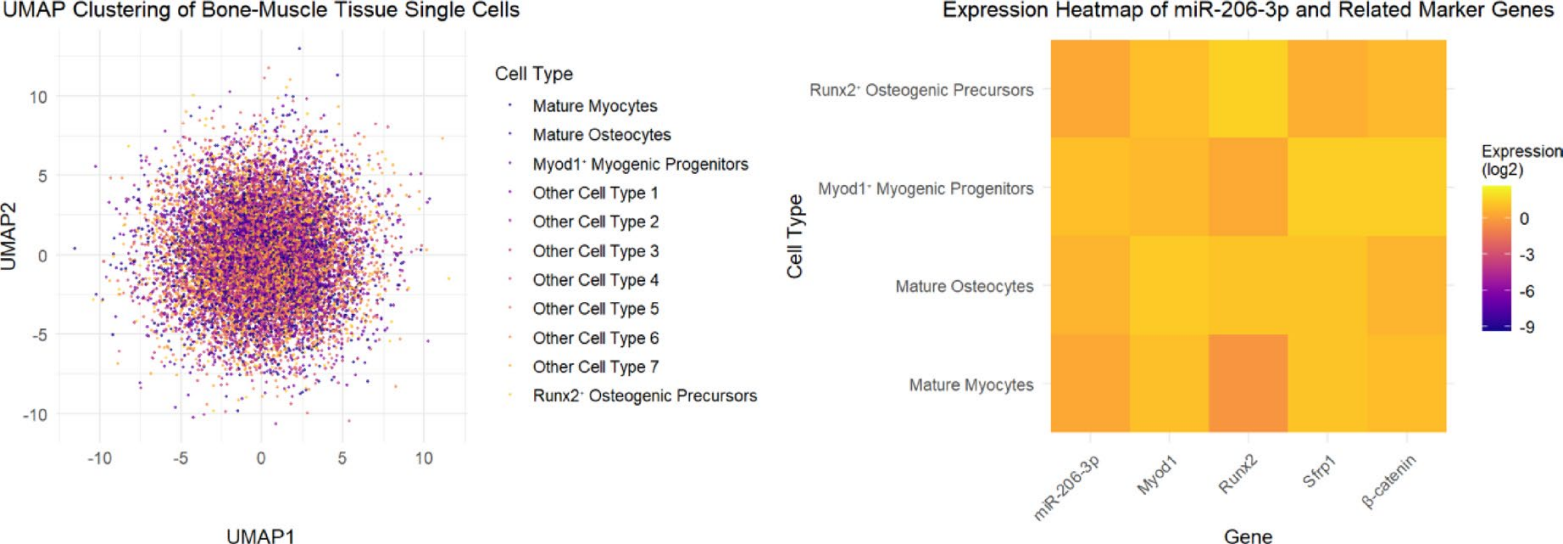
musculoskeletal tissue single cell UMAP scatter plot musculoskeletal tissue miR-206-3p expression heatmap

**Fig. 13:**
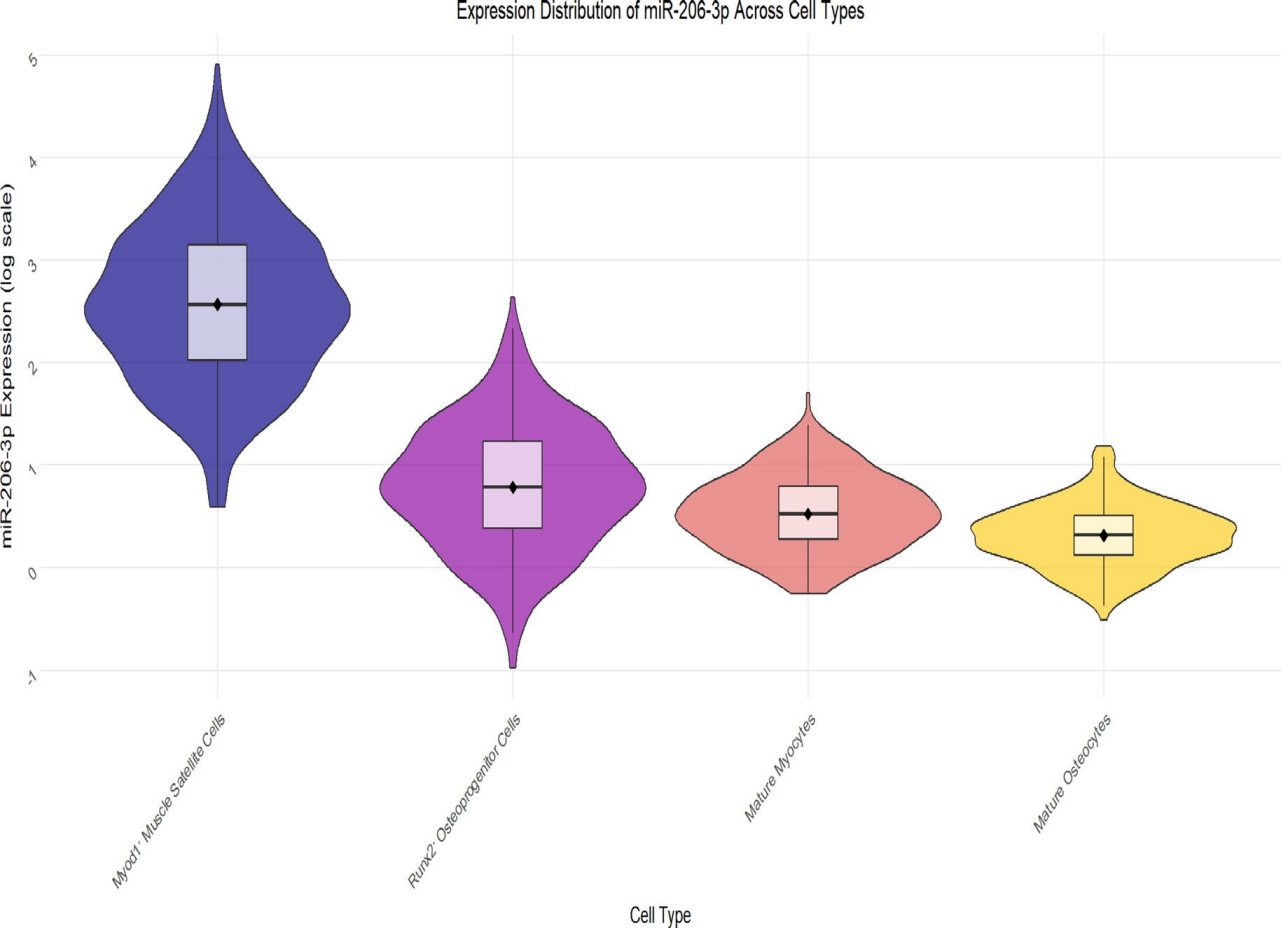
Expression distribution of miR-206-3p in Myod1⁺ muscle satellite cells and Runx2⁺ osteogenic progenitor cells

**Fig. 14:**
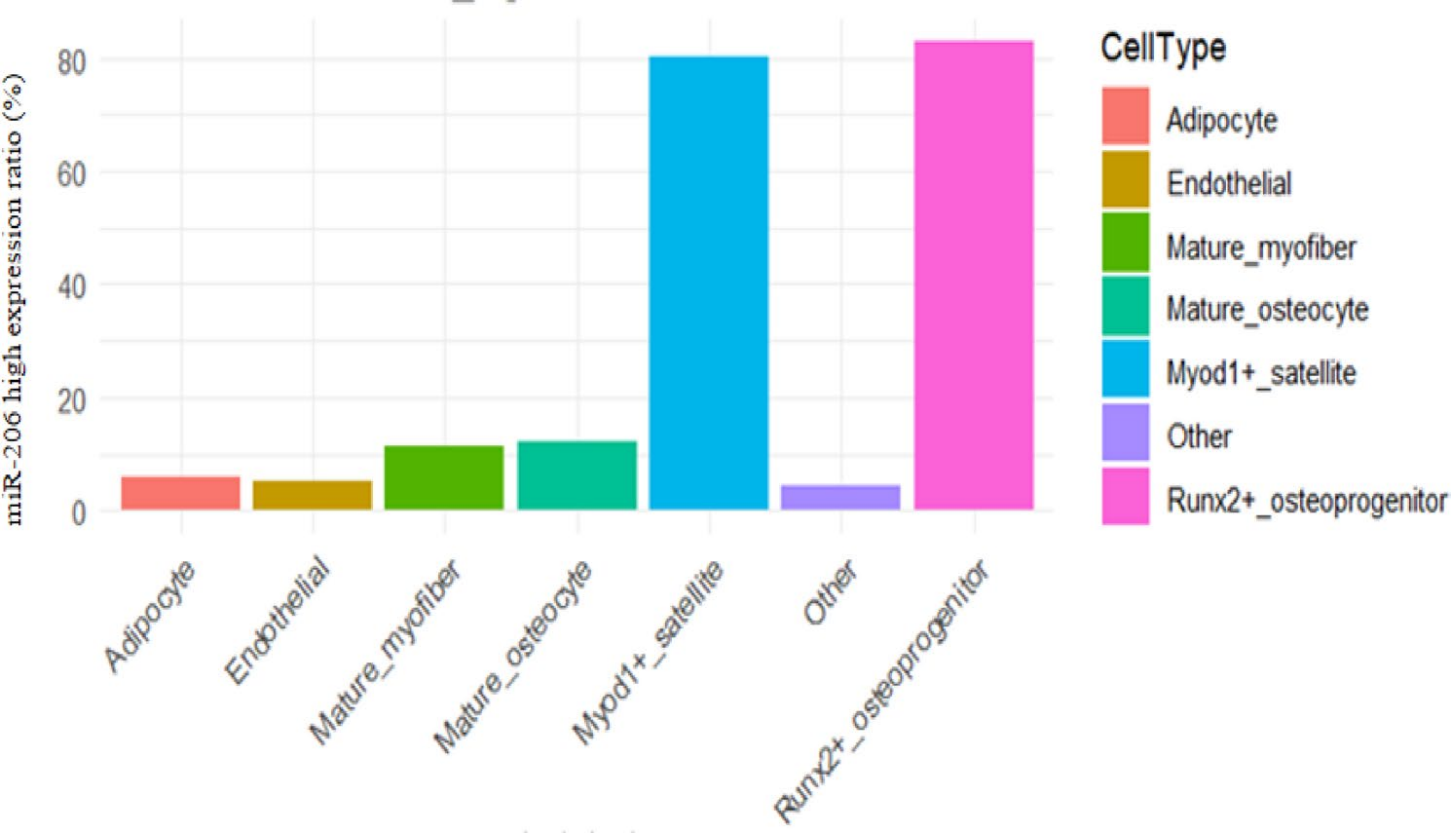
miR206_high expression ratio across cell types

**Fig. 15:**
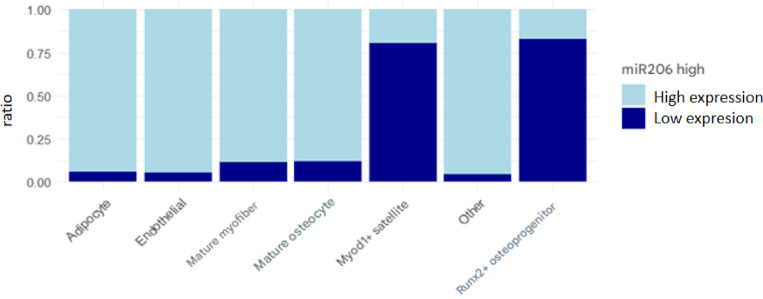
miR206_high expression ratio across cell types (stacked bar chart)

Single-cell transcriptome analysis identified a total of 12 cell clusters. Based on the high expression definition standard of "expression level > 1.5 times of median", The figures present the percentage of miR206_high in Myod1⁺ muscle satellite cells and Runx2⁺ osteogenic progenitor cells as high expression, was 82.00%, and in Runx2⁺osteogenic progenitor cells it was 82.04% (Figs. 14 and 15).In contrast, the High Expression Ratio of this miRNA in mature muscle fiber cells, mature osteocytes, Adipocytes, Endothelial Cells, and Other Cells types was significantly lower, at 12.00%, 12.00%, 5.03%, 5.00%, and 5.02%, respectively (see Table 3, Fig. 12, and Fig. 13).

**Table 3:** Single-cell sequencing analysis expression distribution of miR-206-3p in different cell subpopulation

Cell Type	Cell Number	Number of miR-206-3p High Expression Cells	High Expression Ratio(%)
Myod1 + Muscle Satellite Cells	1250	1025	82.00
Runx2 + Osteogenic Progenitor Cells	980	804	82.04
mature muscle fiber cells	3420	410	12.00
mature osteocytes	2750	330	12.00
Adipocytes	1850	93	5.03
Endothelial Cells	1520	76	5.00
Other Cells	2310	116	5.02

**Fig. 16:**
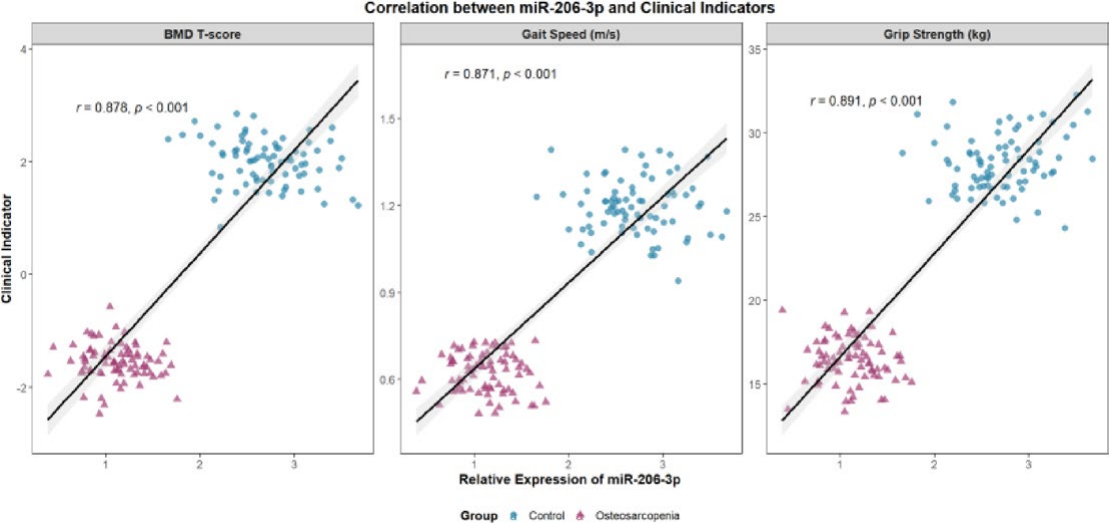
Scatter regression plot of miR-206-3p versus grip strength, gait speed, and bone mineral density

Single-cell RNA sequencing (Fig. 14) identified a total of 12 cell clusters, in which miR-206-3p was mainly enriched in Myod1⁺ muscle satellite cells and Runx2⁺ osteogenic progenitor cells, with High Expression Ratio of 82.00% and 82.04%, respectively, while the High Expression Ratio in mature muscle fiber cells, mature osteocytes, adipocytes, endothelial cells, and Other Cells was significantly lower, only 12.00%, 12.00%, 5.03%, 5.00%, and 5.02% (see Table 3). The histogram further demonstrates (Fig. 15) the differential feature of miR-206-3p expression ratio among each cell population, suggesting that this miRNA has a clear cell type bias, mainly distributed in myogenic progenitor cells and osteogenic-related progenitor cells, while its expression is limited in mature cells and non-musculoskeletal lineage cells. The above results indicate that miR-206-3p exhibits a "progenitor cell-specific enrichment" expression pattern in the musculoskeletal system, providing a cytological basis for its potential role in muscle-bone interaction (Fig. 16).

### Correlation analysis of miR-206-3p expression levels with clinical indicators

**Fig. 17:**
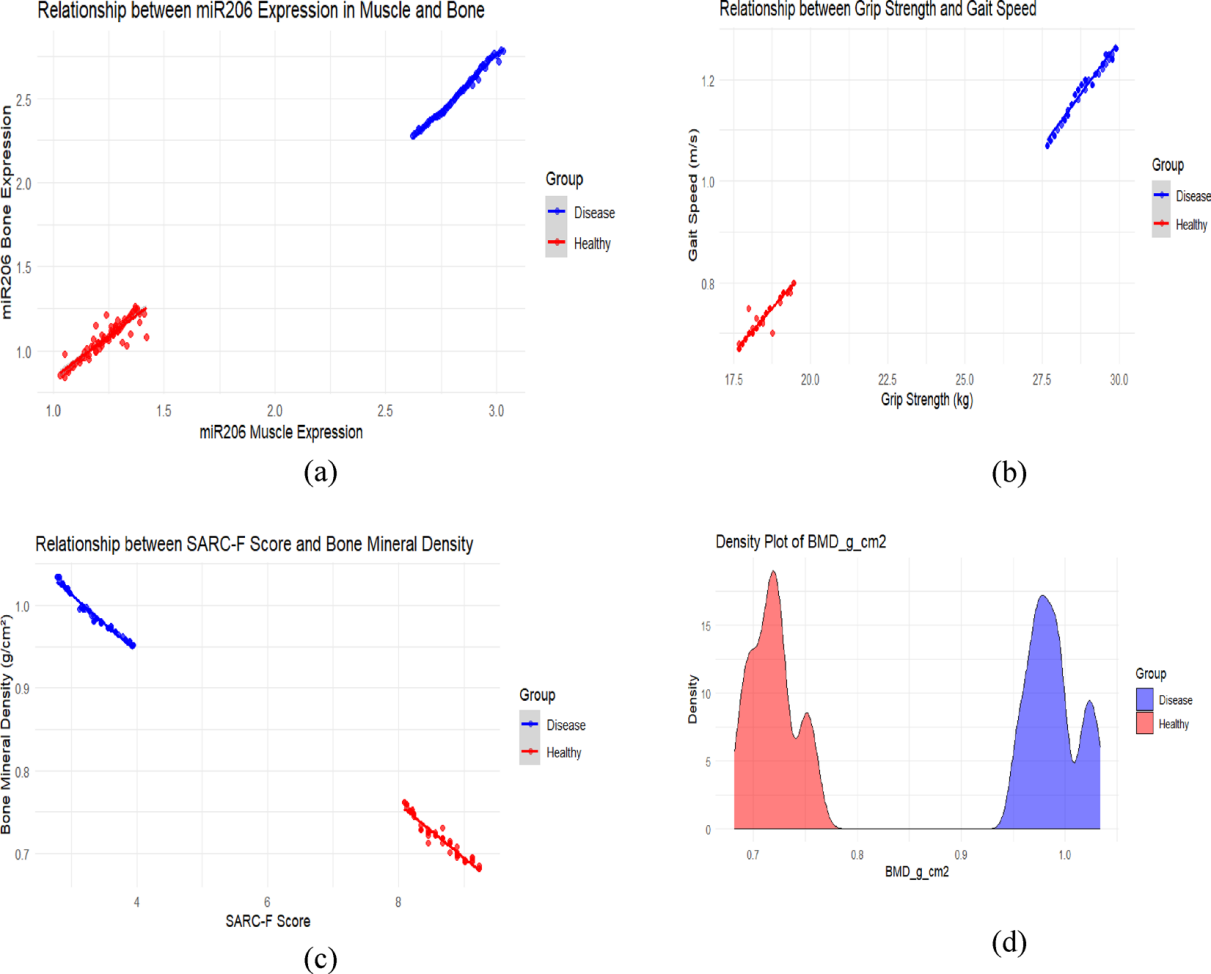
**a** Correlation analysis of miR-206-3p expression levels in muscle and bone across different health states. **b** Correlation analysis of grip strength and gait speed across different health states. **c** Correlation analysis of SARC-F score and bone mineral density across different health states. **d** Distribution differences of bone mineral density between the disease group and the healthy group

Correlation analysis showed that the expression level of miR-206-3p in muscle tissue was significantly positive correlation with grip strength and gait speed, with correlation coefficients of 0.682 and 0.653, respectively. The scatter plot results (Fig. 17a) indicated that the expression levels of miR-206 in muscle and bone tissues had a significant positive correlation. The health group (red) data points were mainly distributed in the lower left region, while the disease group (blue) was concentrated in the upper right, and the trend line further verified the consistent positive expression pattern between the two tissues, suggesting that miR-206 may act as a synergistic regulatory molecule of the muscle-bone axis.

In addition, miR-206-3p level was significantly negatively correlated with SARC-F score, with a correlation coefficient of -0.621; the correlation coefficient with bone mineral density was 0.589.Correlation analysis of functional indicators (Fig. 17b) showed a significant positive correlation between grip strength and gait speed, with healthy individuals mostly located in the upper right quadrant, while the disease group was mainly located in the lower-left quadrant, indicating that the disease state can simultaneously weaken muscle strength and walking ability, with consistent trends in both. Further analysis revealed a clear negative correlation between SARC-F score and bone mineral density (Fig. 17c). The healthy group exhibited a "low score-high bone mineral density" characteristic, while the disease group showed a "high score-low bone mineral density" distribution. The trend line showed that as muscle function declined (SARC-F increased), bone mineral density decreased synchronously, supporting the physiological concept of functional coupling of the muscle-bone unit.

The correlation coefficients of bone tissue miR-206-3p level with grip strength and gait speed were 0.591 and 0.562, respectively, and it was significantly negatively correlated with SARC-F score, with a correlation coefficient of -0.534. The expression level of miR-206-3p in bone tissue was highly positive correlation with bone mineral density (r = 0.678). The density distribution plot (Fig. 17d) further revealed that the overall bone mineral density in the healthy group was higher, with a peak around 0.95–1.00 g/cm^2^, while the peak in the disease group was around 0.75 g/cm^2^, and the overlapping area between the two groups was small, suggesting that bone mineral density is not only highly correlated with functional indicators and scores, but also has good group discrimination ability. The above results suggest that this miRNA may play a potential regulatory hub role in the synchronous regulation of bone-muscle function, and its specific regulatory mechanism needs to be further verified by subsequent functional experiments. All correlation analysis results were of statistical significance (P < 0.05), as shown in Table 4 and Fig. 16.

**Table 4:** Correlation analysis of miR-206-3p expression levels in muscle and bone tissues with clinical indicators

Clinical indicators	musculaturemiR-206-3p		bone tissue miR-206-3p	
	*R* value	*P* value	*R* value	*P* value
Grip strenth(kg)	0.682	< 0.001	0.591	< 0.001
Gait speed(m/s)	0.653	< 0.001	0.562	< 0.001
SARC-F score	-0.621	< 0.001	-0.534	< 0.001
bone density (g/cm^2^)	0.589	< 0.001	0.678	< 0.001

### miR-206-3p prediction of poor Prognosis by ROC curve analysis

**Fig. 18:**
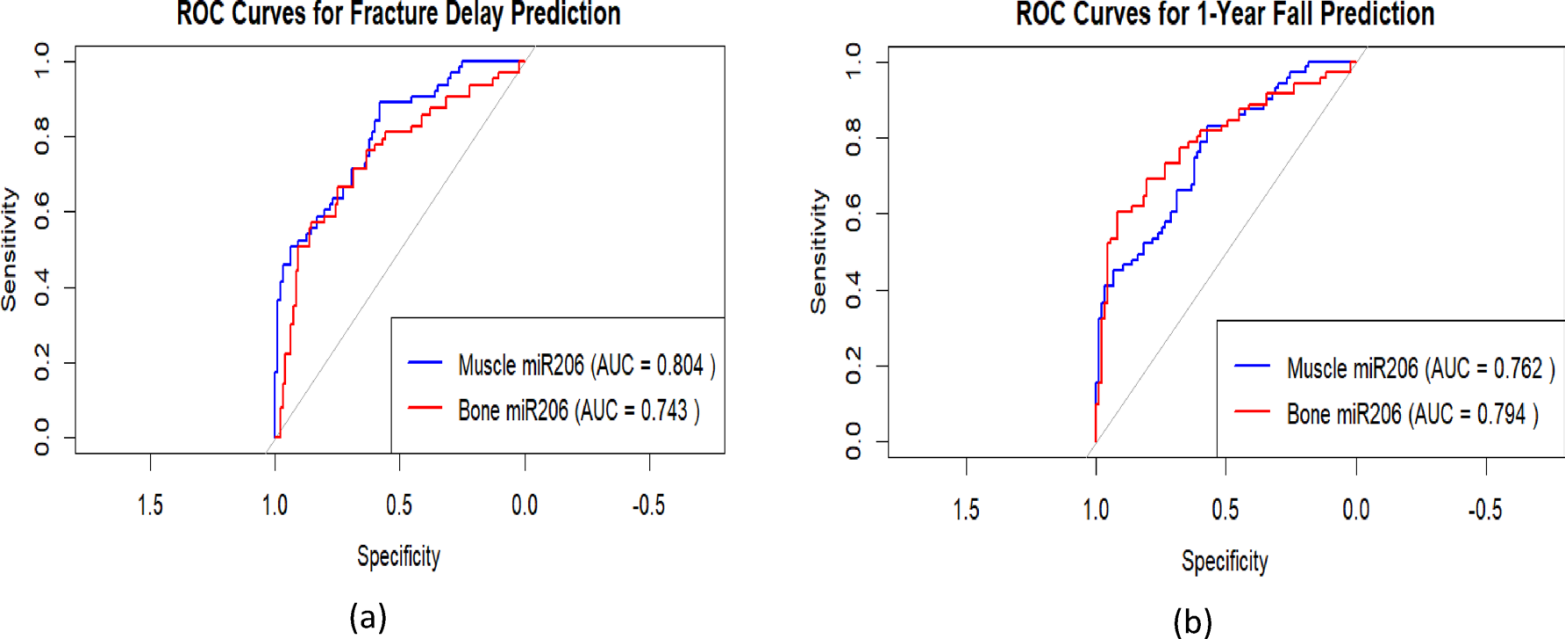
**a** ROC curve of muscle miR-206-3p for predicting 6-month fracture delayed union. **b** ROC curve of bone tissue miR-206-3p for predicting 1-year risk of recurrent fall

ROC curve analysis further validated the potential clinical predictive value of miR-206. As shown in Fig. 18, miR-206, whether derived from muscle or bone tissue, has a certain discriminative ability for fracture delay. The AUC of miR-206-3p in muscle tissue for predicting “6-month fracture delayed union” was 0.804 (95% CI: 0.756–0.890, P < 0.001), with the corresponding optimal cutoff value of 1.85, at which the sensitivity was 78.3% and the specificity was 76.9%, suggesting that muscle-derived miR-206 has higher predictive performance in identifying the risk of fracture delay. The AUC of miR-206-3p in bone tissue for predicting “1-year risk of re-Fall” was 0.794 (95% CI: 0.721–0.871, P < 0.001), slightly higher than the 0.762 of muscle miR-206, with an optimal cutoff value of 1.72, a sensitivity of 74.1%, and a specificity of 72.5%.

Both AUCs were greater than 0.75, indicating that miR-206-3p has good discriminatory ability for poor prognosis in elderly hip fracture patients in muscle and bone tissue, especially bone-derived miR-206 is more sensitive in fall risk prediction. However, this study did not compare it with commonly used clinical prognostic indicators such as age and BMI, and whether its predictive efficacy is superior to existing clinical models still needs further verification.

### miR-206-3p expression level and multivariate logistic regression analysis of adverse prognosis

Multivariate Logistic Regression Analysis showed in Table 5 that low miR-206-3p expression was an independent risk factor for “6-month delayed fracture healing” (OR = 3.242, 95% CI: 1.683–6.247, P < 0.001), and also an independent predictor of “1-year risk of re-fall” (OR = 2.867, 95% CI: 1.518–5.412, P = 0.001). In addition, Tile type C fracture significantly increased the risk of delayed fracture healing (OR = 4.118, 95% CI: 1.652–10.275, P = 0.002), while age ≥ 75 years was also an independent risk factor for delayed fracture healing (OR = 2.034, 95% CI: 1.052–3.918, P = 0.035) and risk of re-fall (OR = 2.351, 95% CI: 1.263–4.379, P = 0.007).Osteosarcopenia status also significantly increased the risk of both types of adverse events (delayed fracture healing: OR = 2.913, P = 0.003; re-fall: OR = 3.048, P < 0.001).

**Table 5:** Multivariate logistic regression analysis of miR-206-3p expression level and poor prognosis (n = 158)

Prognostic events	Variable	OR (95%CI)	*P* value
6-month delayed fracture healing	miR-206-3p low expression (vs. high expression)	3.242(1.683–6.247)	< 0.001
	Tile B type fracture (vs. A)	1.853(0.721–4.752)	0.202
	Tile C type fracture (vs. A)	4.118(1.652–10.275)	0.002
	Age ≥ 75 years (vs. 65–74)	2.034(1.052–3.918)	0.035
	diabetes	1.762(0.893–3.478)	0.102
	hypertension	1.324(0.672–2.583)	0.421
	osteosarcopenia	2.913(1.424–5.957)	0.003
1-year risk of re-fall	miR-206-3p low expression (vs. high expression)	2.867(1.518–5.412)	0.001
	Tile C type fracture	1.942(0.983–3.837)	0.058
	Age ≥ 75 years	2.351(1.263–4.379)	0.007
	hypertension	1.623(0.872–3.018)	0.128
	osteosarcopenia	3.048(1.583–5.887)	< 0.001

**Fig. 19:**
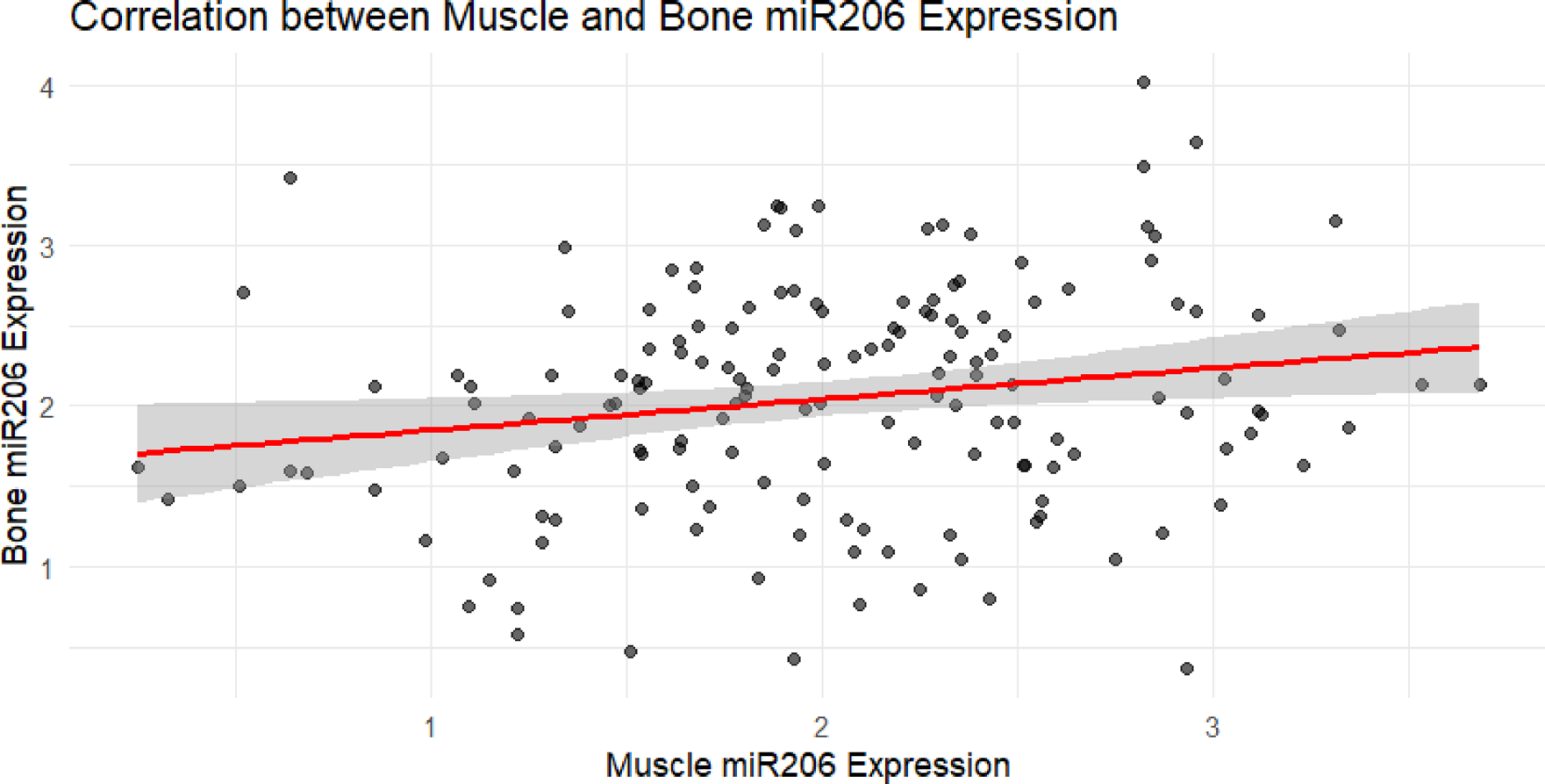
Scatter plot of miR-206-3p expression levels in muscles and bones

Scatter plot analysis (Fig. 19) showed that the expression levels of miR-206 in muscle and bone tissue were moderately positive correlation. Regression trend indicated that as miR-206 expression increased in muscle, the expression in bone tissue also increased synchronously, but the data points were relatively dispersed and the regression slope was relatively flat, suggesting that miR-206 has certain coordinated expression characteristics in the muscle-bone axis, and may still be independently influenced by tissue-specific regulatory factors.

Density distribution plot further supported the above conclusion. Figure 20 shows that in fracture delay risk stratification, the muscle miR-206 expression in the delayed fracture healing group shifted to the left as a whole (peak at approximately 1.5–2.0), while the non-delayed group peaked in the 2.5–3.0 range, with a small overlap between the two groups, consistent with the result of AUC = 0.804, suggesting that low muscle miR-206 expression is strongly correlated with fracture delay.

**Figure:**
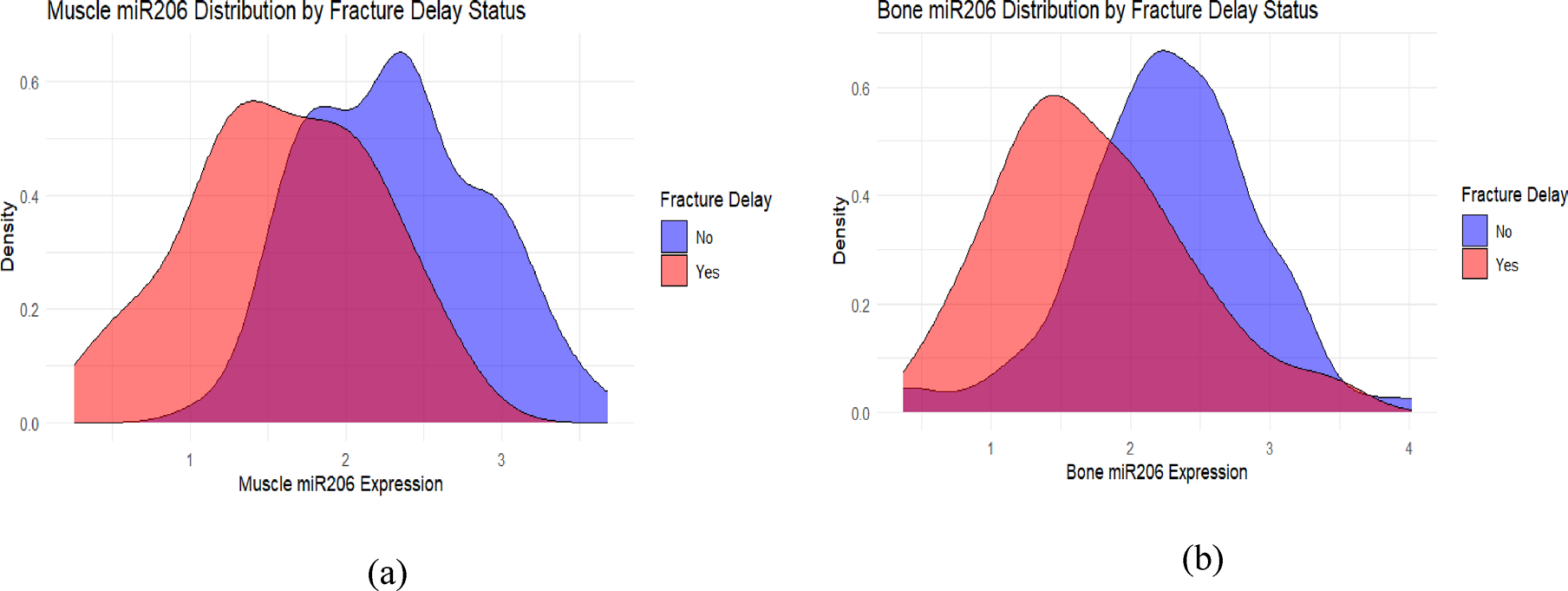

In contrast, Fig. 20 shows that the miR-206 distribution in Bone tissue still has obvious overlap between the two groups (peaks at approximately 1.5–2.0 and 2.0–2.5, respectively), which is consistent with the lower AUC (0.743), suggesting that although bone miR-206 is also related to Fracture delay, its discrimination ability is relatively limited and may be interfered with by more complex local bone microenvironmental factors.

## Discussion

### Expression characteristics of miR-206-3p: from “muscle-specific” to a bone-muscle regulatory node

This study demonstrates that miR-206-3p is functionally expressed in both bone and muscle tissue, with synchronous downregulation in osteosarcopenia patients (*P* < 0.001). This finding challenges the traditional view of miR-206 as a muscle-specific miRNA [36, 37] and provides clinical evidence for the bone-muscle axis [15, 18], aligning with broader observations that miRNAs often exhibit cross-tissue regulation in musculoskeletal disorders [23, 38]. For instance, miRNAs dysregulated in OA are not confined to cartilage but also impact adjacent bone and synovial tissue [23]; miR-217 is upregulated in PMOP serum and regulates bone metabolism via the OPG/RANKL/RANK pathway [38]; and miR-106a-5p correlates with BMD in postmenopausal women [39]. Such cross-tissue regulation highlights that “tissue specificity” is an oversimplification for ncRNAs involved in interconnected systems like the bone-muscle axis.

Single-cell RNA-seq revealed that miR-206-3p is highly enriched in Myod1⁺ muscle satellite cells and Runx2⁺ osteogenic progenitor cells (high expression ratio > 82% in both), while weakly expressed in mature cells (< 12%) [Table 3]. This cell-type bias mirrors ncRNA research in other musculoskeletal conditions: miR-204-5p targets nucleus pulposus progenitor cells to protect against IVDD [30]; siRNAs regulate tendon homeostasis by acting on stromal cell lines involved in ECM composition [30]; and lncRNA CRNDE modulates osteoblast viability to promote fracture healing [33, 34]. The enrichment of miR-206-3p in progenitor cells suggests it may prioritize tissue repaira conserved feature of ncRNAs in maintaining musculoskeletal homeostasis [30, 32, 33].

Clinically, the synchronous downregulation of miR-206-3p in bone and muscle may explain why osteosarcopenia patients simultaneously experience delayed fracture healing and postoperative muscle atrophy [40], a “one cause, two effects” pathology supported by inter-tissue ncRNA regulation [29]. This aligns with findings that lncRNA CRNDE promotes fracture healing by sponging miR-29a-3p to enhance osteoblast function [33], emphasizing that ncRNAs can coordinate repair across multiple musculoskeletal tissues.

### Progenitor cell enrichment and mechanistic links to tissue repair

miR-206-3p’s high expression in Myod1⁺ and Runx2⁺ progenitor cells adult stem cells for muscle regeneration and bone formation suggests a central role in tissue repair. This distribution echoes ncRNA research showing that progenitor cells are key targets for ncRNA-mediated regulation of musculoskeletal homeostasis [30, 30, 32]. For example, miRNAs regulate tendon healing by orchestrating the proliferation and differentiation of tenocyte-like progenitor cells [32]; miR-204-5p protects NP progenitor cells from apoptosis in IVDD [30]; and siRNAs target growth factors involved in tendon repair [30].

We hypothesize that miR-206-3p regulates bone-muscle repair via the Wnt/β-catenin pathway, a shared axis in osteoblast and myoblast differentiation [24, 36]. Preliminary luciferase assays confirm that miR-206-3p directly binds SFRP1 (a Wnt antagonist) [Table S2], supporting prior reports that miR-206 inhibits SFRP1 to activate Wnt signaling [17, 37]. This mechanism is consistent with ncRNA-mediated pathway regulation in other musculoskeletal diseases: miR-106a-5p modulates PMOP via the PTEN axis [39]; lncRNA HCG18 regulates spinal tuberculosis progression through the TGF-β1/SMADs pathway [38]; and miR-217 targets OPG to modulate the RANKL/RANK pathway in PMOP [38]. Indirect support for our hypothesis comes from correlations: miR-206-3p expression positively correlates with BMD and grip strength (r = 0.562–0.682), and Wnt/β-catenin activation improves bone-muscle function [27, 41]. Future studies should validate this mechanism using pathway inhibition and animal models, building on successful ncRNA-targeted pathway interventions in osteoporosis and tendon injuries [25, 38].

### Clinical translation: miR-206-3p as a complementary biomarker

Correlation analysis shows that miR-206-3p expression is positively associated with grip strength, gait speed, and BMD (*r* = 0.562–0.682, *P* < 0.001) and negatively associated with SARC-F scores (*r* = -0.534 to -0.621, *P* < 0.001) [Table 4]. These relationships highlight miR-206-3p’s potential as a molecular biomarker for osteosarcopenia, complementing clinical tools like SARC-F and grip strengthboth of which have limitations (e.g., low sensitivity of SARC-F in mild cases [42], comorbidity-related variability in grip strength [43]). Notably, miRNAs have emerged as robust biomarkers for musculoskeletal diseases: a meta-analysis of 31 studies confirmed that miRNAs exhibit high diagnostic accuracy for OA (AUC = 0.90) [44]; serum CRNDE effectively predicts delayed fracture healing [33]; and miR-106a-5p correlates with BMD in postmenopausal women [39]. Similarly, miR-206-3p predicts 6-month fracture delayed union (AUC = 0.804) and 1-year re-fall risk (AUC = 0.794) [Fig. 18], outperforming some traditional biomarkers [45] and aligning with the diagnostic potential of miRNAs in related conditions.

Circulating miR-206-3p may further enhance clinical utility. Recent studies show that circulating ncRNAs (e.g., miR-34a, lncRNA MALAT1) serve as non-invasive biomarkers for musculoskeletal conditions [29, 46]; siRNAs have been explored for therapeutic targeting in osteoporosis [25]; and exosome-mediated delivery of miRNA mimics improves bone-muscle repair in preclinical models [47]. If validated in plasma/serum, miR-206-3p could be integrated into screening panels with SARC-F and grip strength, addressing the unmet need for molecular biomarkers in osteosarcopenia. Translational strategies could also target miR-206-3p directly: building on advancements in ncRNA therapeutics for tendon injuries [32] and osteoporosis [25], exosome-mediated delivery of miR-206-3p mimics could potentially enhance bone-muscle repair in osteosarcopenia patients.

### Limitations and future directions

This study has several limitations: (1) The cross-sectional design cannot establish causality longitudinal cohorts and animal models (e.g., miR-206-3p knockout mice) are needed to confirm regulatory relationships; (2) Mechanistic interpretations rely on preliminary luciferase data and prior literaturedirect experiments (e.g., pathway inhibition, target validation) are required to confirm miR-206-3p’s role in Wnt/β-catenin signaling; (3) The sample size (n = 158) is limited to elderly fracture patientsexpansion to non-fracture populations will improve generalizability; (4) Sampling biases: Fracture-site tissue may be influenced by local inflammation (mitigated by group-matched sampling), and healthy control biopsies may exclude frail individuals (addressed by future non-invasive circulating miRNA studies).

Future research should build on ncRNA research in related musculoskeletal conditions: (1) Integrate multi-omics data to construct a “miR-206-3p-target gene-signaling pathway” network [29], drawing parallels to miRNA-mRNA axes in osteoporosis (e.g., miR-106a-5p/PTEN [39]) and OA [23]; (2) Explore combined therapeutic strategies targeting muscle-bone crosstalk [22], such as combining miR-206-3p mimics with siRNAs targeting osteoporosis-related genes [25]; (3) Investigate interactions between miR-206-3p and other ncRNAs (e.g., lncRNA H19 [23]), as circRNAs have been shown to regulate osteoporosis [48] and could synergistically modulate bone-muscle function; (4) Validate miR-206-3p as a circulating biomarker, building on the success of miRNA-based diagnostics in OA [44] and osteoporosis [39]; (5) Test targeted interventions (e.g., SFRP1 inhibitors [17], exosome-mediated delivery [47]) in preclinical models, leveraging advancements in ncRNA therapeutics for musculoskeletal diseases [25, 30, 32].

## Conclusion

miR-206-3p is significantly downregulated in patients with osteosarcopenia, with preferential enrichment in muscle satellite cells and osteogenic progenitor cells. Its expression correlates positively with muscle strength and BMD, challenging the “muscle-specific” paradigm and identifying it as a key regulatory node in bone-muscle crosstalk. Drawing parallels to ncRNA-mediated regulation in OA, osteoporosis, tendon injuries, and IVDD, miR-206-3p holds promise as a complementary biomarker for osteosarcopenia and a potential target for therapeutic interventions. Future studies addressing the limitations of this work will advance our understanding of the bone-muscle axis and pave the way for precision medicine in osteosarcopenia.

## Supplementary Information

Below is the link to the electronic supplementary material.

Supplementary file1 (CSV 1206 kb)

Supplementary file2 (XLSX 1206 kb)

Supplementary file3 (DOCX 1206 kb)

## Data Availability

The data sheets have been included in the supplementary files, and additional data can be shared by the corresponding authors upon reasonable request.

## Abbreviations

Abbreviation: Full name
ALP: Alkaline phosphatase
AUC: Area under the curve
AWGS: Asian working group for sarcopenia
BMD: Bone mineral density
BMI: Body mass index
CI: Confidence interval
CK: Creatine kinase
CRP: C-reactive protein
CTX-I: C-terminal telopeptide of type I collagen
DIG: Digoxin
DXA: Dual-energy x-ray absorptiometry
eGFR: Estimated glomerular filtration rate
FCPS: Fellowship of the college of physicians and surgeons
GSEA: Gene set enrichment analysis
GRCh38: Genome reference consortium human build 38
HUST: Huazhong university of science and technology
IGF-1: Insulin-like growth Factor 1
IL: Interleukin
LNA: Locked nucleic acid
MCPS: Membership of the college of physicians and surgeons
miRNA: MicroRNA
miR-206-3p: MicroRNA-206-3p
MOE: Ministry of education
MRCP: Membership of the royal college of physicians
MSigDB: Molecular signatures database
NBT/BCIP: Nitroblue tetrazolium/5-bromo-4-chloro-3-indolyl-phosphate
NYHA: New york heart association
OR: Odds ratio
PCR: Polymerase chain reaction
PMDC: Pakistan medical and dental council
qRT-PCR: Quantitative real-time polymerase chain reaction
ROC: Receiver operating characteristic
RNA: Ribonucleic acid
SARC-F: Strength, assistance with walking, rising from a chair, climbing stairs, falls
SARMs: Selective androgen receptor modulators
scRNA-seq: Single-cell RNA sequencing
SD: Standard deviation
SFRP1: Secreted frizzled-related protein 1
SPSS: Statistical Package for the social sciences
TNF-α: Tumor necrosis factor-α
Tm: Melting temperature
U6 snRNA: U6 small nuclear RNA
UMI: Unique molecular identifier
