# Supplementary material for: The expression characteristics of miR-206-3p in musculoskeletal tissue and its clinical significance
Source: J Orthop Surg Res. 2026 Mar 25;21:293. doi: 10.1186/s13018-026-06785-5 (PMC13141340; doi:10.1186/s13018-026-06785-5)
Supplement: Supplementary file 3 — Supplementary file3 (DOCX 1206 kb) [file 13018_2026_6785_MOESM3_ESM.docx]

TABLE S1: CONSORT-Compliant Recruitment Flow Table

*Supplementary Materials*

| **Step** | **Process Description** | **Number of Participants** | **Details/Exclusion Reasons** |
| --- | --- | --- | --- |
| 1 | Potential Participants (Nov 2022–Dec 2023) | 400 | Elderly hip/proximal femur fracture patients (≥65y, low-energy trauma) + age-matched healthy volunteers (no fracture history) |
| 2 | Preliminary Screening | — | Assess basic inclusion criteria: (1) Age ≥65y; (2) Willing to provide tissue samples; (3) Able to complete grip strength/gait speed/SARC-F/DXA |
| 3 | Meet Basic Inclusion Criteria | 315 | — |
| 4 | Excluded at Preliminary Screening | 85 | - Age <65y (n=14)  - Refusal to provide tissue samples (n=33)  - Inability to complete clinical assessments (n=38) |
| 5 | Comprehensive Eligibility Assessment | — | Evaluate exclusion criteria and confirm clinical data completeness |
| 6 | Excluded at Comprehensive Assessment | 78 | - Other metabolic bone diseases (e.g., hyperparathyroidism; n=15)  - Primary/secondary myopathies (e.g., polymyositis; n=9)  - Malignancy/chemo-radiotherapy (≤6 months; n=11)  - Severe heart/liver/kidney dysfunction (n=22)  - Long-term bone/muscle metabolism-affecting drugs (≥3 months; n=14)  - Missing key clinical/follow-up data (n=7) |
| 7 | Informed Consent Signed | 237 | All participants provided written informed consent (approved by TJ-IRB20221128/TJ-IRB202512049) |
| 8 | Diagnostic Assessments | — | 1. DXA bone densitometry; 2. Grip strength/gait speed tests; 3. SARC-F questionnaire; 4. Comorbidity verification |
| 9 | Group Allocation | — | Based on diagnostic results (osteoporosis: DXA T-score ≤-2.5; sarcopenia: AWGS 2019 criteria) |
| 10 | Osteosarcopenia Group | 79 | Hip fracture + osteoporosis + sarcopenia |
| 11 | Control Group (Non-osteosarcopenia Fracture) | 79 | Hip fracture; T-score >-1.0; normal muscle function |
| 12 | Healthy Control Group | 79 | Age-matched volunteers; no fracture history; T-score >-1.0; normal muscle function |
| 13 | 1-Year Follow-Up Completion | 237 | All participants completed follow-up; outcomes recorded: 6-month fracture delayed union, 1-year re-fall |

Table S1 Legend

CONSORT-compliant recruitment flow table detailing the sequential process of participant screening, eligibility assessment, exclusion, and group allocation. A total of 400 potential participants were initially identified, with 237 advancing to final group allocation after excluding 163 individuals (85 at preliminary screening, 78 at comprehensive eligibility assessment). The three study groups (Osteosarcopenia, Control, Healthy Control) were balanced with 79 participants each, and all completed the 1-year follow-up. This table adheres to CONSORT 2010 guidelines for prospective cohort studies, ensuring transparency in participant selection and traceability of exclusion reasons.

Key CONSORT Compliance Notes

1. **Transparent Enrollment Trajectory**: Explicitly reports participant counts at each stage (potential → screened → eligible → allocated → followed up).
2. **Exclusion Reason Specification**: Details the type and number of exclusions to ensure reproducibility and reduce selection bias.
3. **Alignment with Study Design**: Reflects the three-group structure and age matching for healthy controls, consistent with the manuscript’s methods.
4. **Ethics and Compliance**: Highlights informed consent and IRB approval, aligning with the study’s ethical requirements.
5. **Follow-Up Completion**: Confirms 100% follow-up rate, supporting the reliability of outcome data.

This table complements the CONSORT flow diagram and provides a tabular summary for rapid reference, meeting the *Journal of Orthopaedic Surgery and Research*’s requirements for methodological transparency.

**unified Data Deposition File for***Journal of Orthopaedic Surgery and Research*

This file integrates all 8 CSV datasets (Table 1-1 to Table 5-5) from your osteosarcopenia research, structured to comply with the journal’s mandates for **linked genotype-phenotype data** (core focus) and support other deposited data (e.g., RNA sequencing, miRNA sequences). The file uses standardized formatting, clear metadata, and sample traceability to meet Springer Nature’s research data policy.

**File Overview**

| **Component** | **Content Description** |
| --- | --- |
| **Format** | Multi-sheet Excel file (compatible with repository uploads; CSV version also provided) |
| **Core Data Type** | Linked genotype-phenotype data (miRNA/gene expression + clinical osteosarcopenia phenotypes) |
| **Ethics Compliance** | Aligns with IRB approval: TJ-IRB20221128 (anonymized patient data, no identifiable info) |
| **Repository Fit** | Designed for deposition to **EGA (European Genome-phenome Archive)** (journal-approved for genotype-phenotype data) |

**Sheet 1: Sample Metadata (Merged from Table 1-1, 1-2)**

Unifies sample identification and basic attributes to link all datasets. **Critical for traceability** across molecular and clinical data.

| **Column Name** | **Definition** | **Example Value** |
| --- | --- | --- |
| Sample_ID | Unique anonymized ID for each sample (cross-referenced in all sheets) | OST-001 |
| Tissue_Type | Source tissue for molecular analysis | Bone marrow / Quadriceps muscle |
| Patient_Age | Age of participant (grouped to avoid identification: 60-65y, 66-70y, etc.) | 60-65y |
| Patient_Gender | Gender (coded: M=Male, F=Female) | M |
| Osteosarcopenia_Stage | Clinical stage (based on EWGSOP2 criteria: 0=No, 1=Mild, 2=Severe) | 1 |
| RNA_Seq_Accession | Placeholder for ENA accession (link to deposited RNA sequencing data) | ENA-XXX-XXXX |
| Sample_Quality | RNA integrity score (RIN) for molecular experiments | 8.5 |

**Sheet 2: Molecular Data (Merged from Table 1-3, 1-4, 2-5)**

Links **genotype-related molecular measures** (miRNA/gene expression) to Sample_ID—directly fulfills the journal’s "linked genotype and phenotype data" requirement.

| **Column Name** | **Definition** | **Example Value** |
| --- | --- | --- |
| Sample_ID | Unique ID (matches Sheet 1) | OST-001 |
| miR_206_3p_Expression | Relative expression of miR-206-3p (normalized to U6 snRNA, 2^-ΔΔCt) | 2.15 |
| SFRP1_Expression | Relative expression of target gene SFRP1 (normalized to GAPDH, 2^-ΔΔCt) | 0.82 |
| IL_16_Expression | Relative expression of target gene IL-16 (normalized to GAPDH, 2^-ΔΔCt) | 1.37 |
| Experimental_Method | Technique used for measurement | qRT-PCR (SYBR Green) |
| Batch_Number | Experimental batch (to account for technical variation) | Batch_01 |
| QC_Pass | Whether data passed quality control (Yes/No) | Yes |

**Sheet 3: Clinical Phenotype Data (Merged from Table 3-1, 4-5)**

Links **clinical phenotypes** (osteosarcopenia-related metrics) to Sample_ID—completes the "linked genotype-phenotype" loop.

| **Column Name** | **Definition** | **Example Value** | **Unit** |
| --- | --- | --- | --- |
| Sample_ID | Unique ID (matches Sheet 1/2) | OST-001 | - |
| Grip_Strength | Dominant hand grip strength (measured via dynamometer) | 28.5 | kg |
| BMD_Lumbar | Bone mineral density of L1-L4 vertebrae | 0.89 | g/cm² |
| Muscle_Mass_Index | Appendicular lean mass / height² (ALM/ht²) | 5.8 | kg/m² |
| Fracture_History | Past 5-year fracture history (Yes/No) | No | - |
| Functional_Status | Timed Up and Go (TUG) test result | 12.3 | seconds |
| Phenotype_Measurement_Date | Date of clinical assessment (month/year, no day for anonymity) | 03/2024 | - |

**Sheet 4: Experimental Validation Data (Merged from Table 5-5)**

Includes results of functional experiments (e.g., luciferase assay for miRNA-target binding) to support molecular-phenotype links.

| **Column Name** | **Definition** | **Example Value** |
| --- | --- | --- |
| Experiment_ID | Unique ID for validation experiments | VAL-001 |
| Target_Gene | Gene tested for miRNA binding | SFRP1 |
| miRNA_Treatment | miRNA mimic/inhibitor used (or control) | miR-206-3p mimic |
| Luciferase_Activity | Relative luciferase activity (normalized to Renilla) | 0.42 |
| Statistical_Significance | p-value vs. control group | 0.003 |
| Replicate_Count | Number of biological replicates | 3 |

**Sheet 5: Data Dictionary & Compliance Notes**

Critical for repository reviewers—defines all terms and confirms alignment with journal requirements.

**5.1 Data Dictionary**

- Sample_ID: Anonymized identifier (no link to patient names/IDs) to protect privacy.
- miR_206_3p_Expression: Normalized using the 2^-ΔΔCt method; U6 snRNA = reference gene.
- BMD_Lumbar: Measured via dual-energy X-ray absorptiometry (DXA), per ISCD standards.

**5.2 Journal Compliance Confirmation**

| **Journal Mandate** | **How This File Meets It** |
| --- | --- |
| Linked genotype-phenotype data | Sheets 2 + 3 link miRNA/gene expression (genotype-related) to grip strength/BMD (phenotype) via Sample_ID. |
| Data traceability | Sample_ID unifies all sheets; RNA_Seq_Accession links to deposited sequencing data. |
| Minimum information standards | Data Dictionary defines all columns, units, and experimental methods (e.g., qRT-PCR protocols). |
